# A multi-omics analysis-based model to predict the prognosis of low-grade gliomas

**DOI:** 10.1038/s41598-024-58434-8

**Published:** 2024-04-24

**Authors:** Zhijie Du, Yuehui Jiang, Yueling Yang, Xiaoyu Kang, Jing Yan, Baorui Liu, Mi Yang

**Affiliations:** 1 The Comprehensive Cancer Centre of Nanjing Drum Tower Hospital, Nanjing Drum Tower Hospital Clinical College of Traditional Chinese and Western Medicine, Nanjing University of Chinese Medicine, Nanjing, China; 2 Nanjing Drum Tower Hospital Clinical College of Nanjing University of Chinese Medicine, Nanjing, China; 3 Nanjing Hospital of Chinese Medicine Affiliated to Nanjing University of Chinese Medicine, Nanjing, China; 4 The Comprehensive Cancer Centre of Nanjing Drum Tower Hospital, The Affiliated Hospital of Nanjing University Medical School, Nanjing, China

**Keywords:** Lower-grade gliomas, Prognosis, Prediction, Model, Whole exome sequencing, DNA methylation, Cancer, Computational biology and bioinformatics

## Abstract

Lower-grade gliomas (LGGs) exhibit highly variable clinical behaviors, while classic histology characteristics cannot accurately reflect the authentic biological behaviors, clinical outcomes, and prognosis of LGGs. In this study, we carried out analyses of whole exome sequencing, RNA sequencing and DNA methylation in primary vs. recurrent LGG samples, and also combined the multi-omics data to construct a prognostic prediction model. TCGA-LGG dataset was searched for LGG samples. 523 samples were used for whole exome sequencing analysis, 532 for transcriptional analysis, and 529 for DNA methylation analysis. LASSO regression was used to screen genes with significant association with LGG survival from the frequently mutated genes, differentially expressed genes, and differentially methylated genes, whereby a prediction model for prognosis of LGG was further constructed and validated. The most frequently mutated diver genes in LGGs were IDH1 (77%), TP53 (48%), ATRX (37%), etc. Top significantly up-regulated genes were C6orf15, DAO, MEOX2, etc., and top significantly down-regulated genes were DMBX1, GPR50, HMX2, etc. 2077 genes were more and 299 were less methylated in recurrent vs. primary LGG samples. Thirty-nine genes from the above analysis were included to establish a prediction model of survival, which showed that the high-score group had a very significantly shorter survival than the low-score group in both training and testing sets. ROC analysis showed that AUC was 0.817 for the training set and 0.819 for the testing set. This study will be beneficial to accurately predict the survival of LGGs to identify patients with poor prognosis to take specific treatment as early, which will help improve the treatment outcomes and prognosis of LGG.

## Introduction

Brain and nervous system tumors are fatal tumors that have an estimation of over 300,000 new cases (1.6%) and over 250,000 cancer death (2.5%) globally in 2020^1^. A recent nationwide epidemiological investigation in China revealed that in 2016, there were about 109,000 new cases and 585,000 cancer-death of brain tumor^2^. Malignant primary brain tumors belong to the most difficult-to-treat cancer, with a 5-year overall survival of under 35%^3^. In adults, gliomas are the most common type of malignant brain tumors, making up 80% of all the malignant brain tumors^3,4^, with a universally fatal outcome due to common recurrence and progression. Current routine glioma treatment includes surgery, radiation, chemotherapy, immunotherapy^5^, targeted therapy^6^, etc., which results in unsatisfactory outcomes and prognosis.

Lower-grade gliomas (LGGs) are defined as World Health Organization (WHO) grades II and III gliomas, including diffuse low-grade and intermediate-grade gliomas, accounting for approximately 20% of glioma cases^7^. LGG has a relative better survival compared with high grade glioma, with an average survival of about 7 years^8,9^. However, all LGGs will eventually progress to glioblastoma and death^8^. Due to their highly invasive nature, it is impossible to give complete neurosurgical resection, and thus the residual tumors lead to recurrence and malignant progression to grade IV glioblastomas. LGGs exhibit highly variable clinical behaviors in that some subsets of gliomas remain stable for years while some will quickly progress to glioblastoma (WHO grade IV gliomas) with high extend of malignancy within months. Accurate classification of LGGs based on prediction of prognosis is necessary and paramount for early and precise treatment to achieve satisfactory outcomes and prognosis. However, classic histology characteristics cannot accurately reflect the authentic biological behaviors, clinical outcomes, and prognosis of LGGs.

Genomics identifies mutations related to carcinogenesis and cancer progression. Transcriptomics defines genomics regulation particularly related to hallmarks of cancers^10^. DNA methylation as a major epigenetic signature participates in the regulation of pathogenesis and progression of cancer^11–13^. Some studies employed uni-omics data to predict the prognosis of LGGs, with unsatisfactory results. For example, Kang et al. combined five lncRNAs and three immune cell types to construct a risk model for prediction of survival of LGG^14^. Lin et al. identified differentially expressed lncRNAs in radiosensitive vs radioresistant patients to select and construct a three-lncRNA (contributing to pathogenic processes) signature to predict overall survival of LGGs after radiotherapy^15^. Tan et al. combined six immune associated genes (*CD163, FPR3, LPAR5, P2ry12, PLAUR, SIGLEC1*) to construct a prognostic model for LGG, where the calculated risk score could be used to differentiate the overall survival rates of LGG^16^.

The carcinogenesis and cancer progression are attributable to multiple gene effects^17–19^. Therefore, multiple omics data can more accurately provide clues for the mechanistic studies, prediction of treatment outcomes and prognosis, etc. of cancers. Multi-omics analysis is a more promising way to predict the LGGs prognosis. A study combined analyses of whole exome sequence (WES), DNA copy number, transcriptional analysis (messenger RNA expression, microRNA expression), DNA methylation, and targeted protein expression to classify three nonoverlapping, prognostically significant subtypes of LGGs; IDH mutation and 1p/19q codeletion conferred LGGs most favorable clinical outcomes, and most LGGs without IDH mutation molecularly and clinically exhibited similarity to glioblastoma^20^. Pan et al. developed an i-Modern model, which combined multi-omics, including somatic mutations, copy number variation, transcription profile, miRNA, DNA methylation, etc. with deep learning network to predict the prognosis (high vs low-risk glioma), and thus accurately stratified gliomas patients^21^. However, these studies were in term of all types of gliomas instead of the sole LGGs. Until now, the analysis integrating multi-omics data to predict prognosis of LGGs is still lacking.

In this study, we collected 523, 532, 529 samples of LGGs from TCGA database to carry out analysis of WES, RNA sequencing and DNA methylation to profile the frequently mutated genes, differentially expressed genes (DEGs) and differentially methylated genes (DMGs) in primary vs. recurrent LGG samples and then to combine these multi-omics data to establish a prediction model for the survival of LGGs. This study will be beneficial to accurately predict the survival of LGGs to identify patients with poor prognosis so as to take specific treatment as early, which will be helpful improve the treatment outcomes and prognosis of LGG.

## Results

### WES sequencing

523 samples of LGGs underwent WES sequencing, which showed 504 (96.37%) had mutations in driver genes. The most frequently mutated diver genes were *IDH1* (77%), *TP53* (48%), *ATRX* (37%), *CIC* (22%), *TTN* (11%), *FUBP1* (9%), *PIK3CA* (9%), *NOTCH1* (7%), *MUC16* (6%), *EGFR* (6%), etc. (Fig. S1). Missense mutation was the most common mutations type (Fig. S1). In addition, a few LGG samples showed very high tumoral burden.

**Figure 1:**
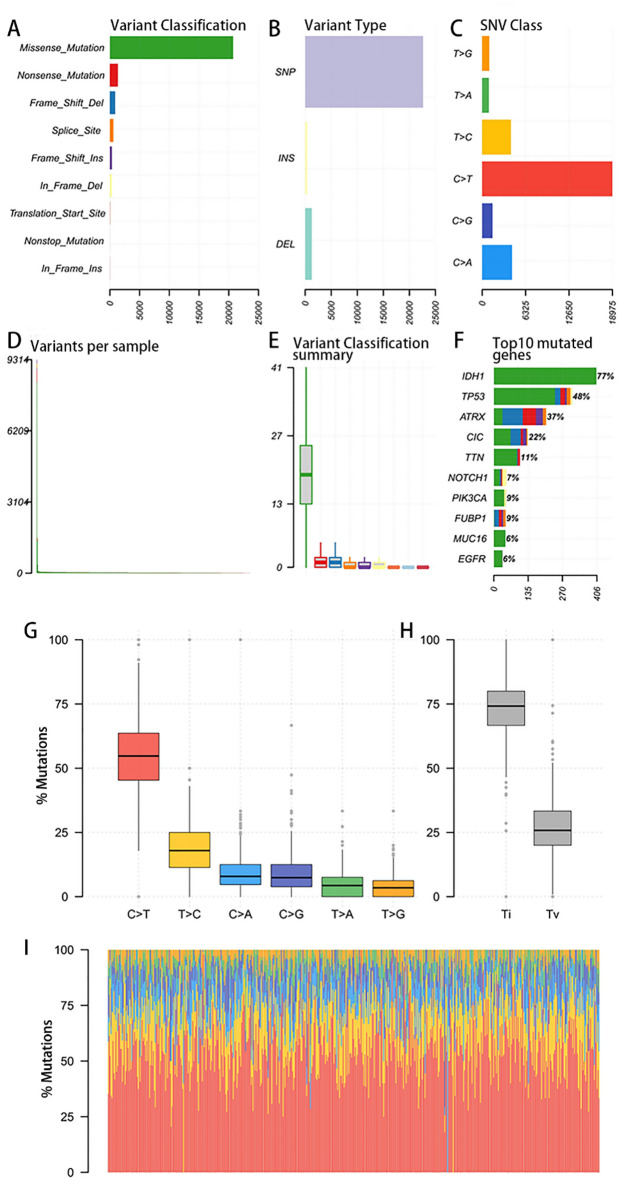
Mutation profile in LGG tissues. (**A**, **B**). the distribution of various variant classification and mutational events (SNP, insertion, and deletion) in all the LGG cases, respectively. (**C**). the number of SNV subtypes (i.e., T > G, T > A, T > C, C > T, C > G, and C > A) in all the LGG cases. (**D**). the average mutation number in each LGG sample. (**E**). the average number of the classified variants in all the LGG cases. (**F**). ten top frequently mutated genes and the components of the involved mutation types. (**G**). the percentages of each type of mutation in LGG samples; (**H**). the percentages of transition (Ti) and transversion (Tv). (**I**). the distribution of mutation types in each LGG sample.

The mutation profile of LGG samples was shown in Fig. 1. Missense mutation was the most common mutations type (Fig. 1A, F–I), and single nucleotide polymorphism (SNP) was the most common variant type among the SNP, insertion (INS) and deletion (Del) in LGG samples (Fig. 1B). For SNV, C > T was the most common subtype (Fig. 1C, G). The mutation number per sample was shown. Normally, the mutation number in LGG patients ranged from tens to hundreds, while a few samples had extremely high mutation number (up to 9000, Fig. 1D, E). The ratios of transition (Ti) and transversion (Tv) were shown, and the Ti percentage was about 75% (Fig. 1H).

The mutation signature in each LGG sample were shown. Among the 30 COSMIC signatures, Signature 1 had the most obvious contribution to mutations in each sample, followed by Signature 14 (Fig. S2A). The correlation heat map about the mutation profile in each sample was shown, where the red and yellow indicated the higher correlation samples while the blue showed the lower correlation samples (Fig. S2B).

**Figure 2:**
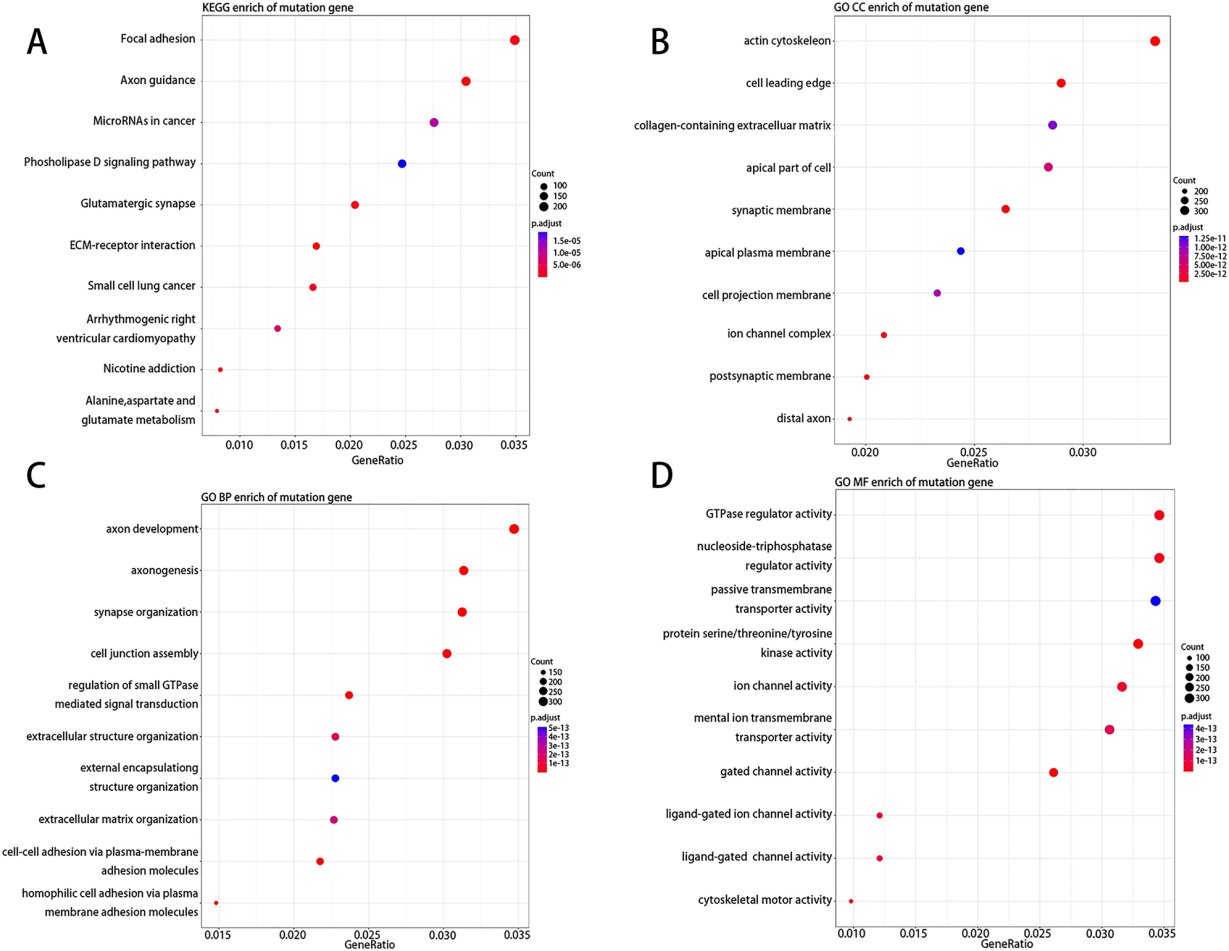
KEGG enrichment analysis (**A**) and GO analysis (**B**–**D**).

KEGG enrichment analysis revealed that the frequently mutated genes were predominantly enriched in neuron function-related signal pathways, such as ‘focal adhesion’, ‘axon guidance’, and ‘glutamatergic synapse’^22^ (Fig. 2A). GO analysis showed that the frequently mutated genes were enriched in actin cytoskeleton, cell leading edge, etc. by cellular component (CC) enrichment, in axon development, axonogenesis, synapse organization, cell junction assembly, etc. by biological process (BP) enrichment, in GTPase regulator activity, nucleoside-triphosphatase regulator activity, etc. by molecular function (MF) enrichment (Fig. 2B–D).

### RNA sequencing

Recurrence is an important characteristics of tumor malignancy, therefore, we observed the transcriptomics in 514 primary versus 18 recurrent LGG tissues through RNA sequencing. The differentially expressed RNAs with adjusted *p* values (padj) less than 0.05 and log2 fold change > 2 were selected. The significantly up- and down-regulated genes in recurrent vs. primary LGG tissues were shown in Tables S1and S2, most of which were protein-coding genes, and the remaining were mostly lncRNAs. Top 50 significantly up-regulated DEGs were *C6orf15, DAO, MEOX2, LINC02587, LTF, MIR3976HG, LINC00507, LINC00588, LGR6, LINC02822,* etc. (Table 1). Top 50 significantly down-regulated DEGs were *DMBX1, GPR50, HMX2, MAFA, COL2A1, TLX3, KC877982.1, BARX1, LBX1-AS1, MEOX1,* etc. (Table 2).

**Table 1:** Top 50 significantly up-regulated genes in recurrent versus primary LGGs.

chr	gene_name	gene_type	padj	log2FoldChange
1	C6orf15	protein_coding	0.020647	4.630677
2	DAO	protein_coding	8.50E − 11	4.55818
3	MEOX2	protein_coding	2.00E − 08	4.380905
4	LINC02587	lncRNA	6.22E − 06	4.266166
5	LTF	protein_coding	2.62E − 07	4.072193
6	MIR3976HG	lncRNA	2.47E − 05	4.063753
7	LINC00507	lncRNA	1.08E − 05	3.921326
8	LINC00588	lncRNA	0.001003	3.903372
9	LGR6	protein_coding	1.30E − 07	3.78286
10	LINC02822	lncRNA	0.000116	3.667197
11	SRY	protein_coding	0.014441	3.647781
12	LINC02475	lncRNA	0.000164	3.442847
13	AL355596.2	lncRNA	7.60E − 05	3.411305
14	TESPA1	protein_coding	1.04E − 05	3.345157
15	KRT6A	protein_coding	0.016291	3.313301
16	LINC01876	lncRNA	0.003923	3.307712
17	NPBWR2	protein_coding	0.004894	3.295289
18	KRT13	protein_coding	0.012046	3.252798
19	AC021613.1	lncRNA	2.30E − 05	3.147213
20	LIPF	protein_coding	0.045985	3.142065
21	CTXN3	protein_coding	0.000474	3.136516
22	AC025160.1	lncRNA	0.014267	3.131171
23	AL450352.1	lncRNA	0.010436	3.090237
24	CARTPT	protein_coding	0.002573	3.043549
25	LINC01007	lncRNA	0.007918	3.031537
26	PCDHGB5	protein_coding	3.89E − 06	3.010299
27	SMCP	protein_coding	0.000137	2.997794
28	SLC17A8	protein_coding	3.40E − 06	2.995641
29	ITPRID1	protein_coding	0.000497	2.992869
30	PAX3	protein_coding	0.021307	2.982502
31	LINC01055	lncRNA	0.002868	2.966434
32	AL355916.2	lncRNA	3.71E − 10	2.964165
33	LINC00898	lncRNA	0.006861	2.953068
34	AC008708.2	lncRNA	0.000578	2.920835
35	AC112236.1	lncRNA	0.000101	2.917577
36	LINC02470	lncRNA	0.001243	2.913919
37	TEKT1	protein_coding	3.37E − 07	2.9087
38	KCNS1	protein_coding	3.32E − 05	2.895573
39	SHISAL2B	protein_coding	0.000194	2.860025
40	PRSS16	protein_coding	0.000123	2.858637
41	HOXA7	protein_coding	0.021428	2.848676
42	AL158065.1	lncRNA	0.000985	2.845254
43	AC092447.5	lncRNA	0.003189	2.839307
44	AP003032.1	lncRNA	0.036997	2.834437
45	SLC14A2	protein_coding	2.54E − 07	2.832973
46	DSG3	protein_coding	0.006162	2.832559
47	AC023421.1	lncRNA	6.20E − 07	2.81472
48	ANKRD34C	protein_coding	3.97E − 05	2.773477
49	AGR3	protein_coding	0.039242	2.771957
50	AC079584.1	lncRNA	0.001088	2.723869

**Table 2:** Top 50 significantly down-regulated genes in recurrent versus primary LGGs.

chr	gene_name	gene_type	pvalue	padj
1	DMBX1	protein_coding	5.99E − 24	4.28E − 20
2	GPR50	protein_coding	4.95E − 20	1.18E − 16
3	HMX2	protein_coding	4.51E − 07	2.82E − 05
4	MAFA	protein_coding	7.84E − 30	1.40E − 25
5	COL2A1	protein_coding	1.86E − 26	2.21E − 22
6	TLX3	protein_coding	2.20E − 06	9.78E − 05
7	KC877982.1	lncRNA	5.58E − 11	1.63E − 08
8	BARX1	protein_coding	5.74E − 16	8.19E − 13
9	LBX1-AS1	lncRNA	1.09E − 07	9.18E − 06
10	MEOX1	protein_coding	1.26E − 22	5.62E − 19
11	AL596442.3	lncRNA	6.32E − 07	3.68E − 05
12	HES2	protein_coding	1.75E − 20	5.19E − 17
13	IGF2	protein_coding	5.15E − 18	9.66E − 15
14	ONECUT3	protein_coding	1.14E − 23	6.78E − 20
15	BNC1	protein_coding	1.81E − 24	1.62E − 20
16	AC120498.10	lncRNA	1.05E − 17	1.74E − 14
17	LMNTD2-AS1	lncRNA	1.30E − 30	4.62E − 26
18	HOXA11-AS	lncRNA	1.90E − 06	8.69E − 05
19	PITX1	protein_coding	8.39E − 14	5.65E − 11
20	IGF2-AS	lncRNA	4.33E − 12	1.86E − 09
21	AC078906.1	lncRNA	3.37E − 14	2.56E − 11
22	AC209154.1	lncRNA	0.001841	0.015912
23	H2AC13	protein_coding	1.33E − 15	1.52E − 12
24	ALOX15	protein_coding	2.76E − 16	4.10E − 13
25	COL3A1	protein_coding	9.45E − 14	6.24E − 11
26	PRND	protein_coding	2.52E − 12	1.18E − 09
27	COL1A1	protein_coding	2.04E − 12	9.83E − 10
28	AC126175.2	lncRNA	1.41E − 12	7.07E − 10
29	AC004835.1	lncRNA	1.49E − 14	1.33E − 11
30	NTF3	protein_coding	3.12E − 12	1.39E − 09
31	HOXB8	protein_coding	0.001643	0.014601
32	AC004080.2	lncRNA	2.56E − 05	0.000646
33	GATA6-AS1	lncRNA	3.42E − 07	2.29E − 05
34	NKX3-2	protein_coding	3.90E − 10	9.28E − 08
35	ROR2	protein_coding	2.92E − 14	2.32E − 11
36	TBX1	protein_coding	1.22E − 19	2.72E − 16
37	EBF2	protein_coding	2.49E − 15	2.61E − 12
38	H3C10	protein_coding	3.53E − 20	9.00E − 17
39	COMP	protein_coding	6.35E − 11	1.81E − 08
40	MAFA-AS1	lncRNA	4.60E − 05	0.000999
41	AC026310.2	lncRNA	8.85E − 11	2.48E − 08
42	MFAP2	protein_coding	1.56E − 11	5.46E − 09
43	COL5A1	protein_coding	4.12E − 12	1.79E − 09
44	HOXC5	protein_coding	0.00033	0.00441
45	CDX2	protein_coding	4.94E − 07	3.03E − 05
46	GP2	protein_coding	0.001194	0.011494
47	MMP9	protein_coding	8.20E − 07	4.51E − 05
48	MNX1	protein_coding	1.34E − 08	1.62E − 06
49	SLC22A31	protein_coding	5.12E − 09	7.58E − 07
50	HES7	protein_coding	2.25E − 20	6.18E − 17

**Figure 3:**
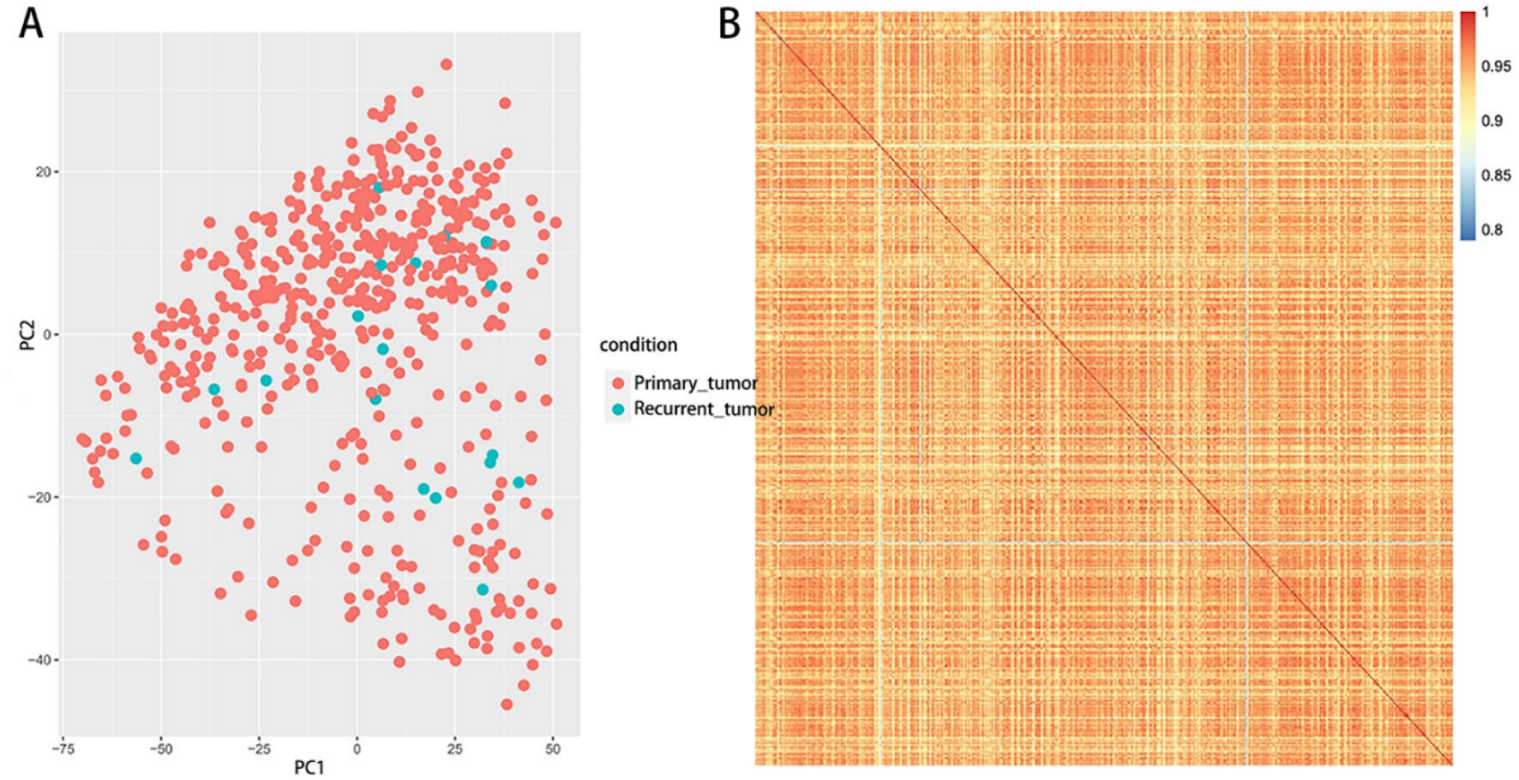
The inter-sample PCA dimension-reduction cluster analysis of the gene expression in primary and recurrent samples. (**A**). plot based on the first and second primary components (PC1 and PC2) showed the gathering among the primary (shown in red) and recurrent tumor (shown in blue) tissues. (**B**). the correlation matrix across all samples according to the gene expression of each sample, whereby the heat map of the correlation matrix after clustering was mapped.

The relationship between the RNA samples from the primary and recurrent LGG tissues was primarily analyzed through the inter-sample PCA dimension-reduction cluster analysis. A plot was profiled based on the first and second primary components (PC1 and PC2) to show the gathering among samples (Fig. 3A). The correlation matrix across all samples according to the gene expression of each sample, whereby the heat map of the correlation matrix after clustering was mapped (Fig. 3B).

The Trans Per Million (TPM) and Fragments Per Kilobase Million (FPKM) of recurrent vs. primary LGG tissues were shown in Fig. S3. The distribution of the expression in primary and recurrent samples were similar, without obvious bias, which meant that they were both suitable for the subsequent analysis. Particularly, TPM is more suitable for comparison between these LGG samples due to the standardization of sequencing depth by TPM.

**Figure 4:**
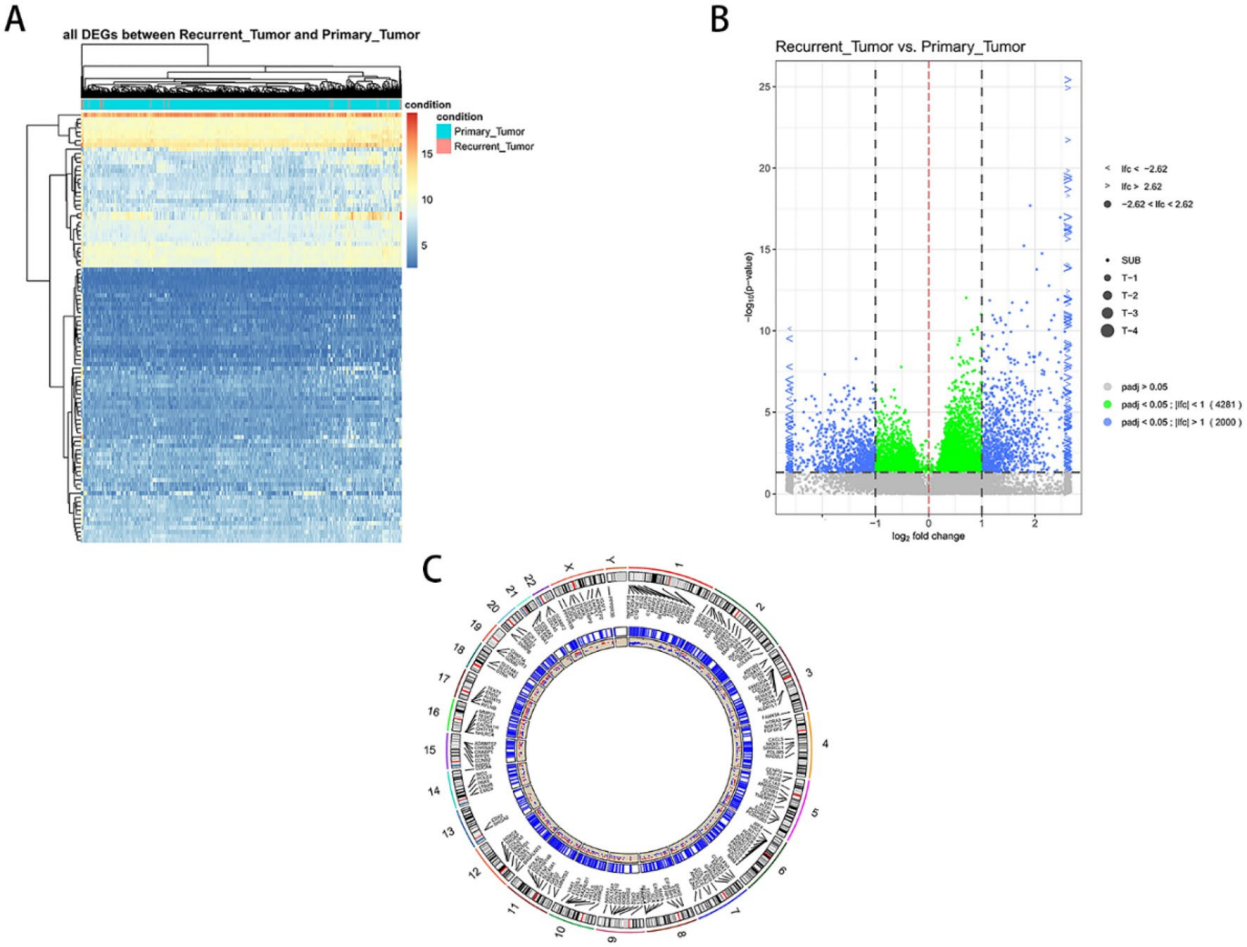
DEGs in LGG tissues and the genomic location. (**A**). Heat-map of the DEGs in LGG tissues. (**B**). Volcano Plot of the DEGs in LGG tissues. (**C**). The genomic location of the DEGs.

Heat-map of DEGs showed the primary LGG samples were mainly clustered in the middle and the recurrent samples were in both sides (Fig. 4A). Volcano Plot showed DEGs with padj < 0.05 and log2 fold change > 2 (Fig. 4B). The chromosomal distribution analysis showed that the recurrence-related DEGs were lest aggregated in Chromosome 13 (Fig. 4C).

**Figure 5:**
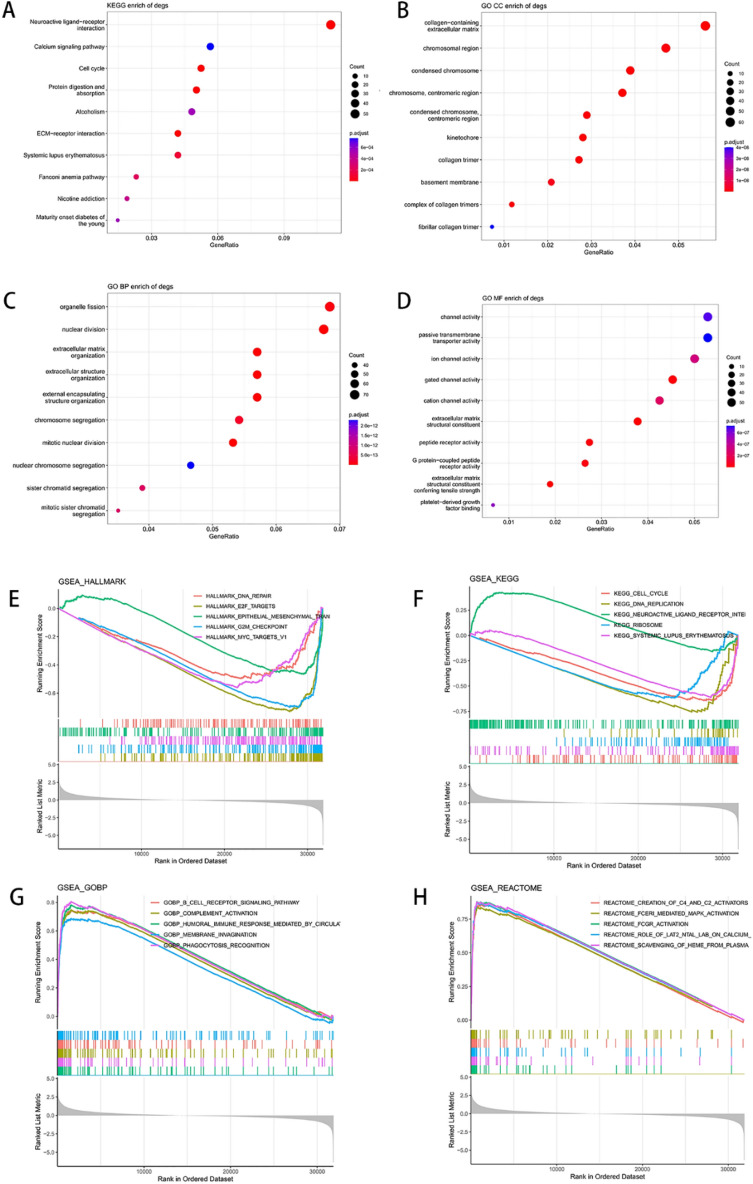
Functional enrichment of differentially expressed genes by KEGG and GO analysis.

Functional enrichment analysis revealed that the DEGs were mainly enriched in neuroactive ligand-receptor interaction by KEGG enrichment^23^ (Fig. 5A), in collagen-containing extracellular matrix, chromosomal region, condensed chromosome, etc. by GO-CC enrichment (Fig. 5B), in organelle fission, nuclear division, extracellular matrix organization, etc. by GO-BP enrichment (Fig. 5C), and in channel activity, passive transmembrane transporter activity, etc. by GO-MF enrichment (Fig. 5D). In addition, the general expression variation trend of the entries in each sample in four databases of HALLMARK, KEGG, GO-BP and REACTOME underwent GSEA analysis. It could be seen that the enrichment pathways in HALLMARK and KEGG mainly exhibited a down-regulation trend, while those in GO-BP and REACTOME mainly showed an up-regulation trend (Fig. 5E–H).

### DNA methylation

**Figure 6:**
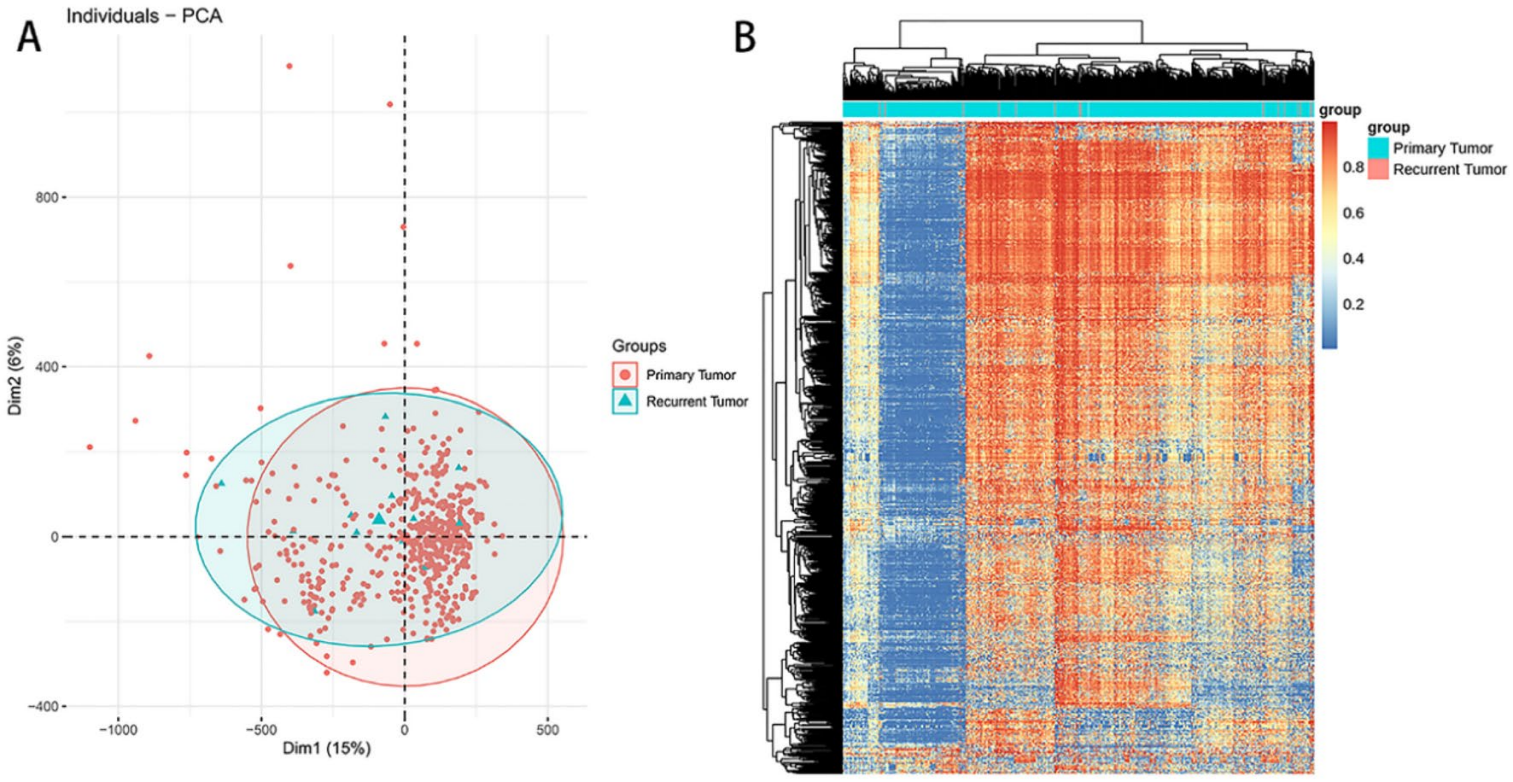
RNA analysis within the LGG tissues. (**A**). PCA dimension-reduction analysis showed some profile difference between recurrent and primary samples. (**B**). The heatmap of the differential methylation of each sample in recurrent versus primary samples.

After exclusion of the samples with incomplete information, 529 samples were analyzed for DNA methylation, including 515 primary and 14 recurrent LGG tissues. PCA dimension-reduction analysis demonstrated some profile difference between the recurrent and primary samples (Fig. 6A). The heatmap of the differential methylation of each sample in recurrent vs. primary LGG samples were shown (Fig. 6B).

Volcanic plot of differential methylation between the primary and recurrent LGG samples showed 2077 genes were more methylated and 299 were less methylated in recurrent vs. primary samples (Fig. S4A). The distribution analysis showed the differential methylation probes were mainly within genes (including gene body and IGR) (Fig. S4B).

**Figure 7:**
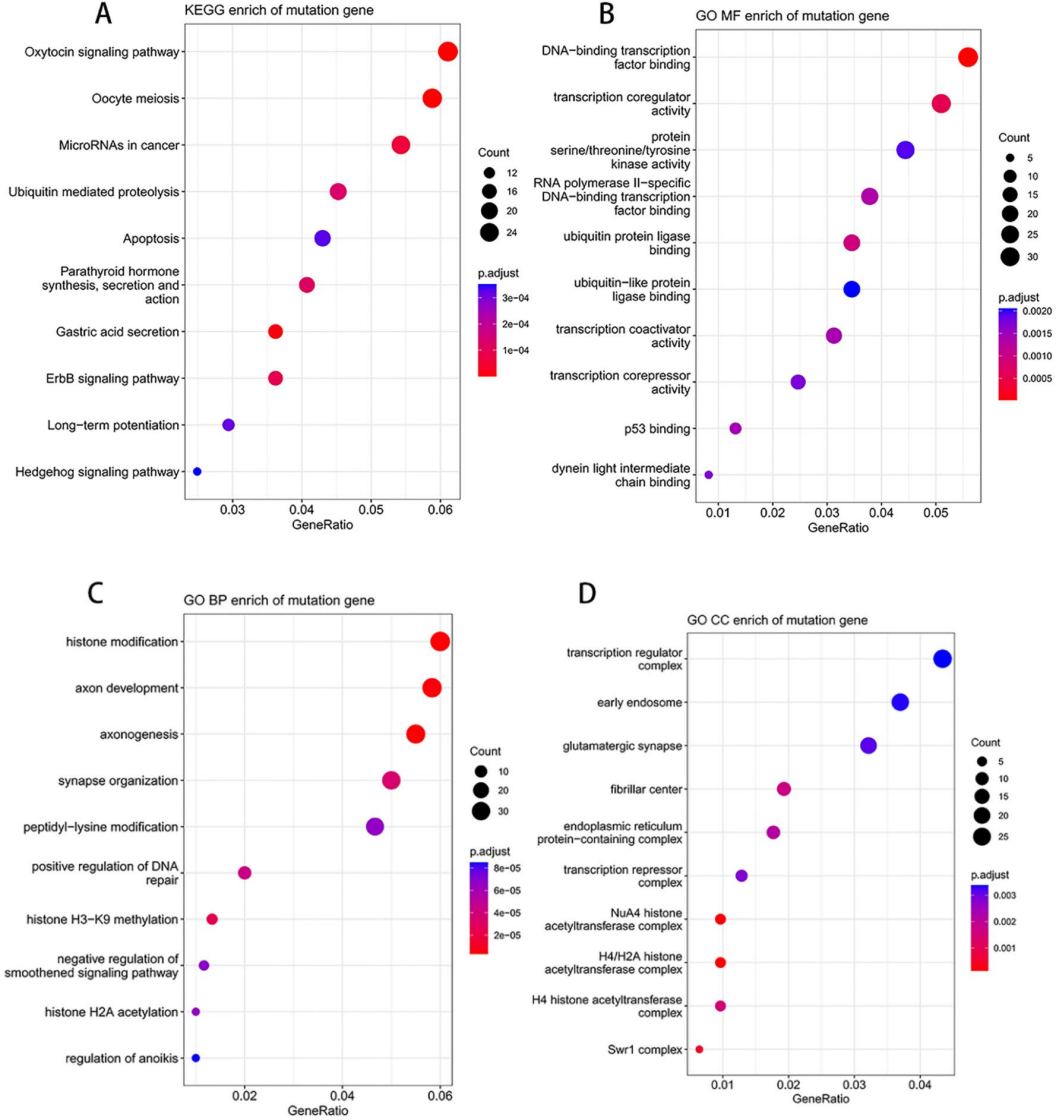
Enrichment analysis of the DME by KEGG (**A**) and GO (**B**–**D**) analysis, respectively.

The DMEs were mainly enriched in neuroactive ligand-receptor interaction, cAMP signaling pathway, focal adhesion, axon guidance, etc. by KEGG enrichment^24^, in protein serine/threonine/tyrosine-kinase activity, channel activity, passive transmembrane transportor activity, protein serine/threonine kinase activity, etc. by GO MF enrichment, in axon development, axonogenesis, synapse organization, regulation of neuron projection development, etc. by GO BP enrichment, and in presynapse, cell- cell junction, collagen-containing extracellular matrix, synaptic membrane, etc. by GO CC enrichment (Fig. 7).

### Prediction model for LGG survival

LASSO analysis showed that among the frequently mutated genes, *IDH2, CIC, IDH1, MUC16, EGFR* and *SMARCA4* were most associated with LGG survival. These gene were further used to build a model, which showed that in the training set, high model score group had a very significant shorter survival than the low-score group (*p* < 0.0001), with a ROC AUC of 0.715 (Fig. S5A,B), which was validated in the testing set, with a ROC AUC of 0.721 (Fig. S5C,D).

Among the DEGs, 22 genes, including *BNC1, LINC02587, HOXC4, H2BC12, IGF2BP2, ABCC3, HMX1, AGMO, OTP, CMYA5, WEE1, MND1, AL390755.1, TNFRSF11B, SCNN1B, SRY, IRX5, HOXA7, LINC01965, ISL2, DMRTA2,* and *IGFBP2* were most associated with LGG survival. A model combination of these DEGs showed in the training set, high model score group had a very significant shorter survival than the low-score group (*p* < 0.0001), with a ROC AUC of 0.859 (Fig. S6A,B), which was validated in the testing set, with a ROC AUC of 0.765 (Fig. S6C,D).

DMEs analysis by LASSO showed that *KIAA1598, TUBA1B, WEE1, PARK2, CRYGD, PDGFB, E2F2, GLT25D2, BDNFOS, DHX36,* and *SRRM4* methylation was significantly associated with LGG survival. A model combining these DMEs indicated that in the training set, the high model score group had a very significantly shorter survival than the low-score group (*p* < 0.0001, Fig. S7A), which was also found in the testing set (Fig. S7C). The ROC analysis showed an AUC of 0.742 for training set (Fig. S7B) and 0.722 for testing set (Fig. S7D).

**Figure 8:**
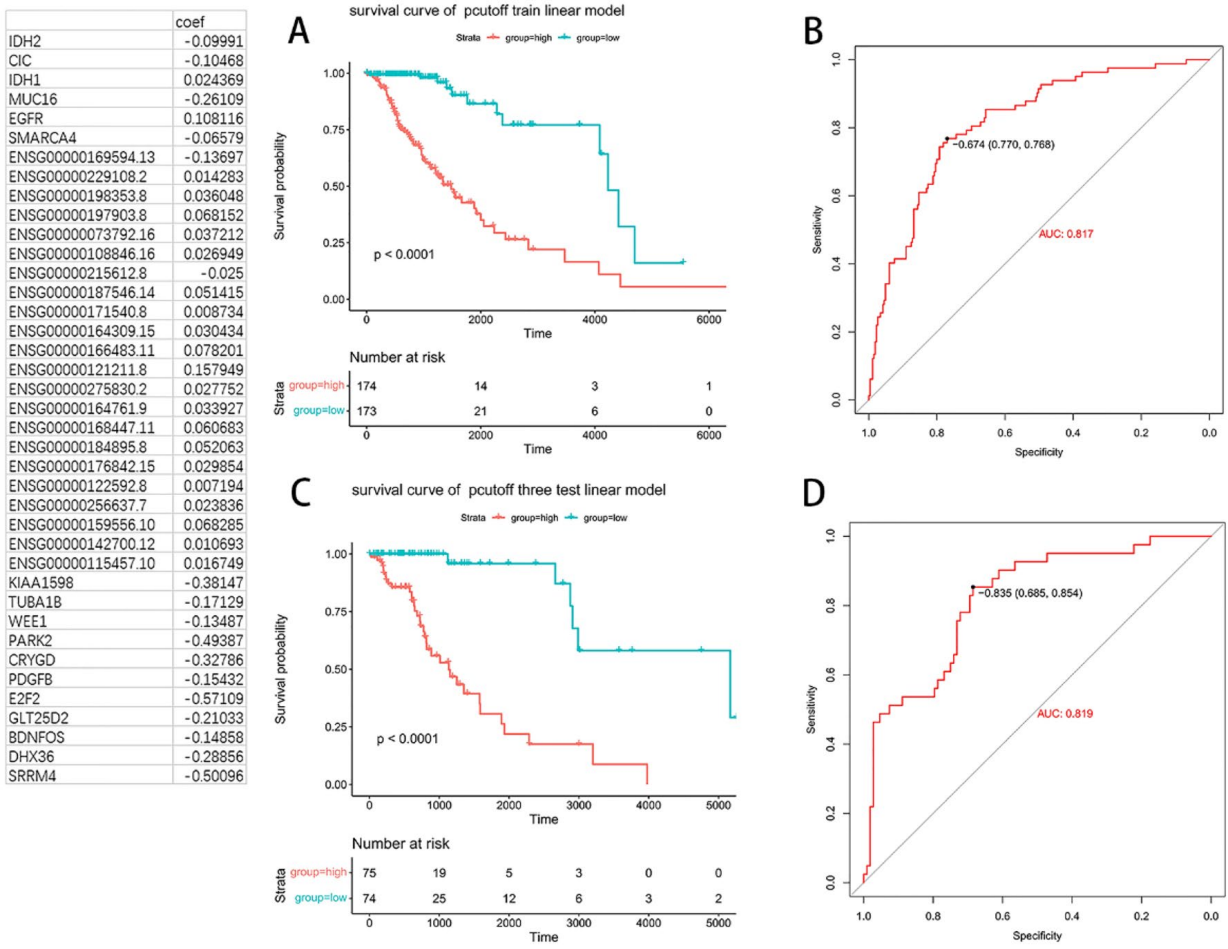
K–M survival analysis and ROC analysis for 39 genes-based model in the training and testing sets. K-M survival analysis in the training (**A**) and testing (**C**) set, respectively. ROC analysis in the training (**B**) and testing (**D**) set, respectively.

Eventually, 39 genes from the analysis of genomic, RNA sequencing and DNA methylation were combined to establish a prediction model for survival of LGG patients. In the training set, the high model score group had a very significantly shorter survival than high-score group (*p* < 0.0001, Fig. 8A). ROC analysis showed that AUC for the model was 0.817 (Fig. 8A–B). Similar result was observed in the testing set, with an AUC for ROC analysis of 0.819 (Fig. 8C–D).

### Establishment of nomogram prognostic model

After constructing the risk scoring model, we developed single-omics and multi-omics Nomogram prognostic models to analyze the predictive capabilities of various feature genes for prognosis. The Nomogram model from the single-omics data revealed, consistent with clinical outcomes, that *IDH2* was the most powerful predictor in the WES model (Fig. S8A). In the transcriptomics model, the most influential genes were *BNC1* (ENSG00000169594.13) and *AGMO* (ENSG00000187546.14) (Fig. S8C). For the methylation model, *SRRM4* emerged as the strongest predictor (Fig. S8E). Upon integrating information from all three omics, the genes with the highest predictive capabilities were the transcriptomic expressions of *WEE1* (ENSG00000166483.11), *BNC1*, and *AGMO* (Fig. S8G). Survival ROC analysis for both single-omics and multi-omics showed that, similar to the risk scoring model, integrating the three omics data improved the accuracy and stability of the predictive models (Figs. S8B, S8D, S8F and S8H).

## Discussion

The carcinogenesis and cancer progression are related to comprehensive effects of multiple genes. Multi-omics analysis is a more promising way to predict the LGGs prognosis. However, the analysis integrating multi-omics data to predict prognosis of LGGs is still lacking. In this study, we collected LGGs samples from TCGA database to carry out analysis of WES, transcription, and DNA methylation and then to combine these multi-omics data to establish a prediction model for the survival of LGGs. This study will be beneficial to accurately predict the survival of LGGs to identify patients with poor prognosis to take specific treatment as early, which will be helpful improve the treatment outcomes and prognosis of LGG.

Mutations in *IDH1* characterize the majority of LGG, which is associated with a favorable prognosis^25^. Consistently, we observed a high mutation frequency (77%) in *IDH1*. *ATRX* is also a frequently mutated gene. Liu et al. detected 33% *ATRX* mutations in grade II gliomas and 46% in grade III gliomas–grade II and III gliomas belong to LGG^26^. Similarly, we reported similar mutation frequency (37%) in *ATRX*. In addition, we observed frequent mutations in *TP53* (48%), *CIC* (22%), *TTN* (11%), et al. This result will be beneficial to explore the mechanisms of the carcinogenesis and progression of LGG as well as the prevention and management of LGG.

For the WES data, we showed a few samples had extremely high (up to over 9000) mutation number, while normally the mutation number in LGG patients ranged from tens to hundreds. This suggests very high TMB in some LGG cases, which might be helpful to the personalized treatment for LGG patients.

Through RNA sequencing, we showed most significantly up-regulated recurrence-related DEGs included *C6orf15, DAO, MEOX2, LINC02587, LTF, MIR3976HG, LINC00507, LINC00588, LGR6, LINC02822*, etc., and most significantly down-regulated DGEs included *DMBX1, GPR50, HMX2, MAFA, COL2A1, TLX3, KC877982.1, BARX1, LBX1-AS1, MEOX1,* etc. This result would provide clues to the mechanistic study on the recurrence of LGG.

Notably, we showed that the recurrence-related DEGs were lest aggregated in Chromosome 13. This implies that the upstream and downstream regulation of DEGs might be lost in genes locating in Chromosomal 13, suggesting the potential roles of Chromosomal 13 genes in the negative regulation of recurrence-related DEGs in LGG.

DNA methylation is a major epigenetic signature occurring in early cancer events, which exhibits great potential in the risk assessment, early identification and prognosis prediction of various cancers^27–29^. We showed 2077 genes were more methylated and 299 were less methylated in recurrent vs. primary samples, and that the differential methylation was mainly within genes, which might suggest the epigenetic alterations in recurrence of LGG.

We combined the screened 6 survival-related frequently mutated genes, 22 DEGs, and 11 DMEs to build a prediction model for LGG survival, which showed the potential to differentiate the subjects with shorter survival from those with longer survival in both the training and testing sets. In addition, the AUC for the combined model was higher than that for WES-, RNA sequencing or DNA methylation-based model, indicating the advantages of the multi-omics data-based model in the prediction of prognosis of LGG.

One limitation of this study is the use of relatively simple linear regression to construct predictive models. We also attempted to use DeepOmix^30^, a deep learning framework for multi-omicsdata, to construct predictive models, but encountered severe overfitting issues (Fig S9). While employing simple models helps to some extent in mitigating overfitting issues due to a limited sample size, it also constrains the model’s ability to improve accuracy with an increasing number of future samples. In future research, employing deep learning methods optimized for addressing overfitting issues would be a preferable option^31^.

This study analyzed the LGG samples from the TCGA database, where the genetic characteristics and clinical variables had some difference from those in Chinese population. Next, tissue samples will be collected from the Chinse LGG patients to validate this result.

## Materials and methods

### Data collation

The GEO and TCGA datasets were searched, while only TCGA-LGG dataset had a adequate sample number (more than 500) and simultaneously had WES, RNA and methylation data sets, which met the requirement of multi-omics modeling analysis. Therefore, the TCGA-LGG dataset was finally selected for subsequent analysis. The TCGAbiolinks R package was used to download WES and RNAseq data, and the processed methylation signal matrix data was downloaded from UCSC XENA. 523 samples were used for WES analysis, 532 for transcriptional analysis and 529 for DNA methylation analysis (based on signal values on 450 K methylation chip).

### Multi-omics analysis

The downloaded WES data were kept as Maf files. The Maftools R was used for data reading, mutation information statistics, and oncoplot mapping, MutationPatters R package for signature analysis, and ClusterProfiler R package for functional enrichment analysis.

For the downloaded RNAseq read count data, the DESeq2 R package was used for data reading, standardization, and differential expression analysis. The vidger R package was used for mapping the volcano plot, the RCircos R package for screening the distribution of DEGs on the genome. Gene Ontology (GO) and Kyoto Encyclopedia of Genes and Genomes (KEGG) functional enrichment analysis^22–24^ of DEGs was performed using the ClusterProfiler R package. The important pathways of MsigDB, KEGG, GO and REACTOME databases were enriched by GSVA R package.

For the downloaded methylation data, ChAMP R package was used for data reading, standardization, and calculation of the differential methylation probe and DMEs. Principal component analysis (PCA) dimension-reduction analysis was performed using the FactoMineR and factoextra R packages. Pheatmap and ggplot R packages were used to draw the related images. Functional enrichment analysis of DMEs was performed using the ClusterProfiler R package.

### Multi-level modeling analysis

As illustrated in the flowchart (Fig S10), we initially applied LASSO for feature selection on the WES, transcriptomic, and methylation data from the TCGA-LGG dataset. Subsequently, the selected feature genes were employed to independently construct both single-omics and multi-omics prediction models.

In the feature screening stage, the highly frequently mutated genes, DMGs, and DMGs obtained in the above uni-omics analysis were evaluated. The LASSO method by glmnet R package was used to conduct feature selection with the survival status as the target values and the mutation, expression change folds of DEGs and methylation change folds of DMGs as the characteristic values. When using the LASSO model for feature selection, the alpha parameter of glmnet was set to 1, and the family parameter was set to ‘cox’. The minimum lambda of LASSO model was referred to screen the characteristic genes from each omics data. After that, each omics data-based survival model was constructed, using survival R package to group samples based on the median risk values of each sample and carry out the corresponding Kaplan–Meier (KM) survival analysis. The pROC R package was used to plot the Receiver Operating Characteristic Curve (ROC) and perform the area under the curve of ROC (AUC) calculations. The characteristic gene data of the above three omics were combined to build the multilevel model.

The relationship between the frequently mutated genes (with mutation frequency of greater than 10%), DEGs and DMGs and patient survival was calculated. Both single-omics and multi-omics models were built using samples containing the three types of omics data mentioned above. Before training the model, 30% of the samples were randomly selected from the dataset as the test set, and the remaining 70% of the samples were used for training. Then, the genes from each kind of omics data were selected to build a survival model based on the respective omics data. Finally, the genes selected from the three kinds of omics were combined to build a multi-omics survival model based on the three kinds of omics data. When training both single-omics and multi-omics survival models, the alpha parameter of glmnet was set to 0, the family parameter was set to ‘cox’, and the nfolds parameter was set to 10 to implement cross-validation. To evaluate the performance of each model, the trained model-related risk scores were calculated and used to observe the correlation with the survival, and ROC curve analysis was carried out to evaluate the performance of the model in the training and test data sets.

To further analyze the indicative role of the selected feature genes on prognosis as previously reported^32^, we employed the rms R package. We constructed both univariate and multi-omic Nomogram prognostic models for the feature genes identified in WES, transcriptome, and methylation datasets. Additionally, we utilized the survivalROC R package to assess the accuracy of each Nomogram model in predicting 1-year, 2-year, and 5-year survival rates.

## Conclusion

We carried out WES, RNA sequencing and DNA methylation analysis to build a multi-omics-based model for prediction of the survival of LGG. This study will be useful to accurately predict the survival of LGGs to take specific treatment for those with poor expected prognosis as early, which will be helpful improve the treatment outcomes and prognosis of LGG.

### Supplementary Information

Supplementary Information.

## Data Availability

All data generated or analyzed during this study are included in this published article.
