# Supplementary material for: A multi-omics analysis-based model to predict the prognosis of low-grade gliomas
Source: Sci Rep. 2024 Apr 24;14:9427. doi: 10.1038/s41598-024-58434-8 (PMC11043340; doi:10.1038/s41598-024-58434-8)
Supplement: Supplementary file 1 — Supplementary Information. [file 41598_2024_58434_MOESM1_ESM.docx]

**
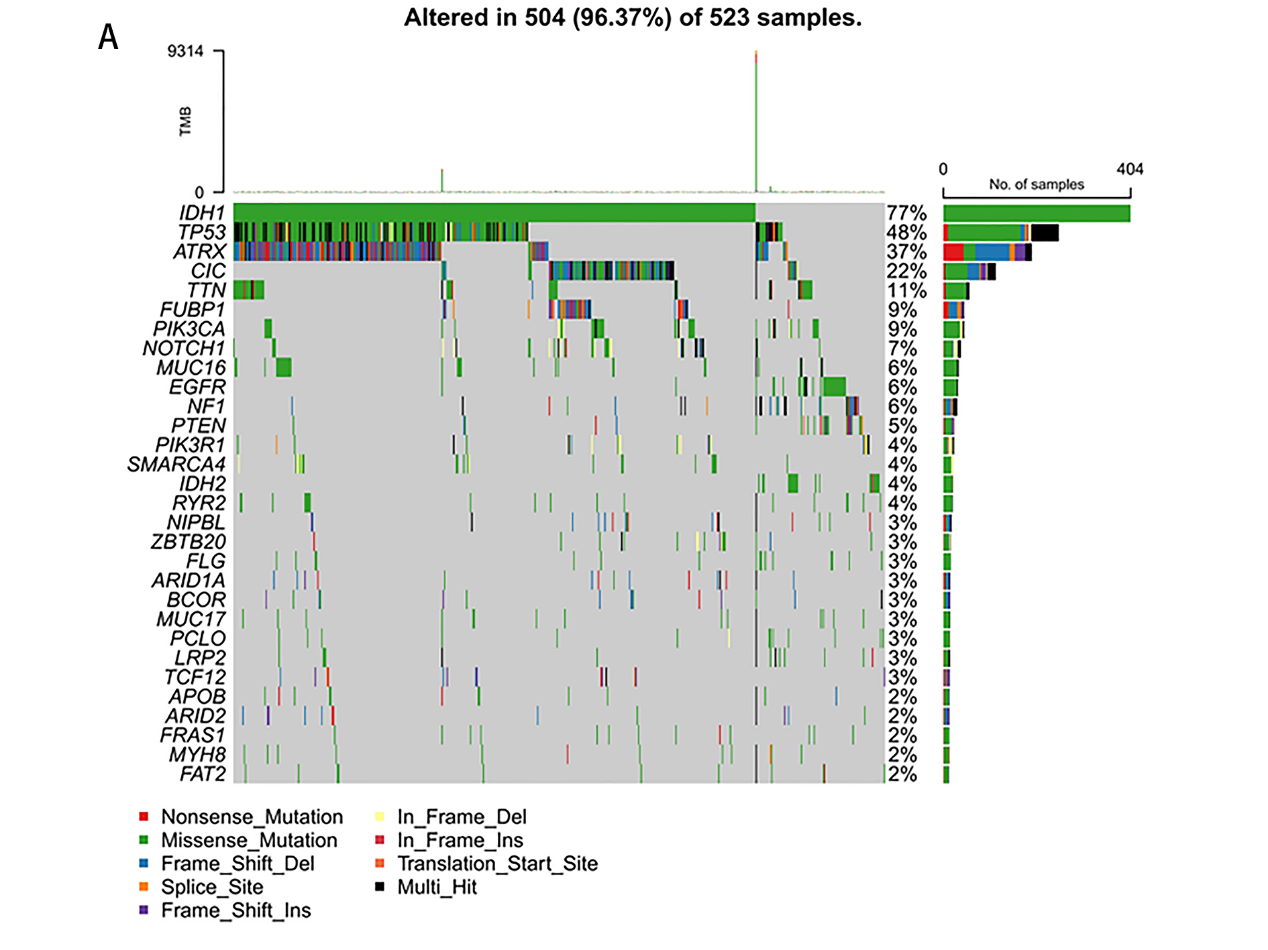
**

**Figure S1 Frequently mutated genes in LGG samples.** Left upper, tumoral burden in each LGG sample; left bottom, frequently mutated genes in the left axis, with the corresponding mutation frequency in the right axis. Right, the number of samples and mutation types for each frequently mutated gene.

**
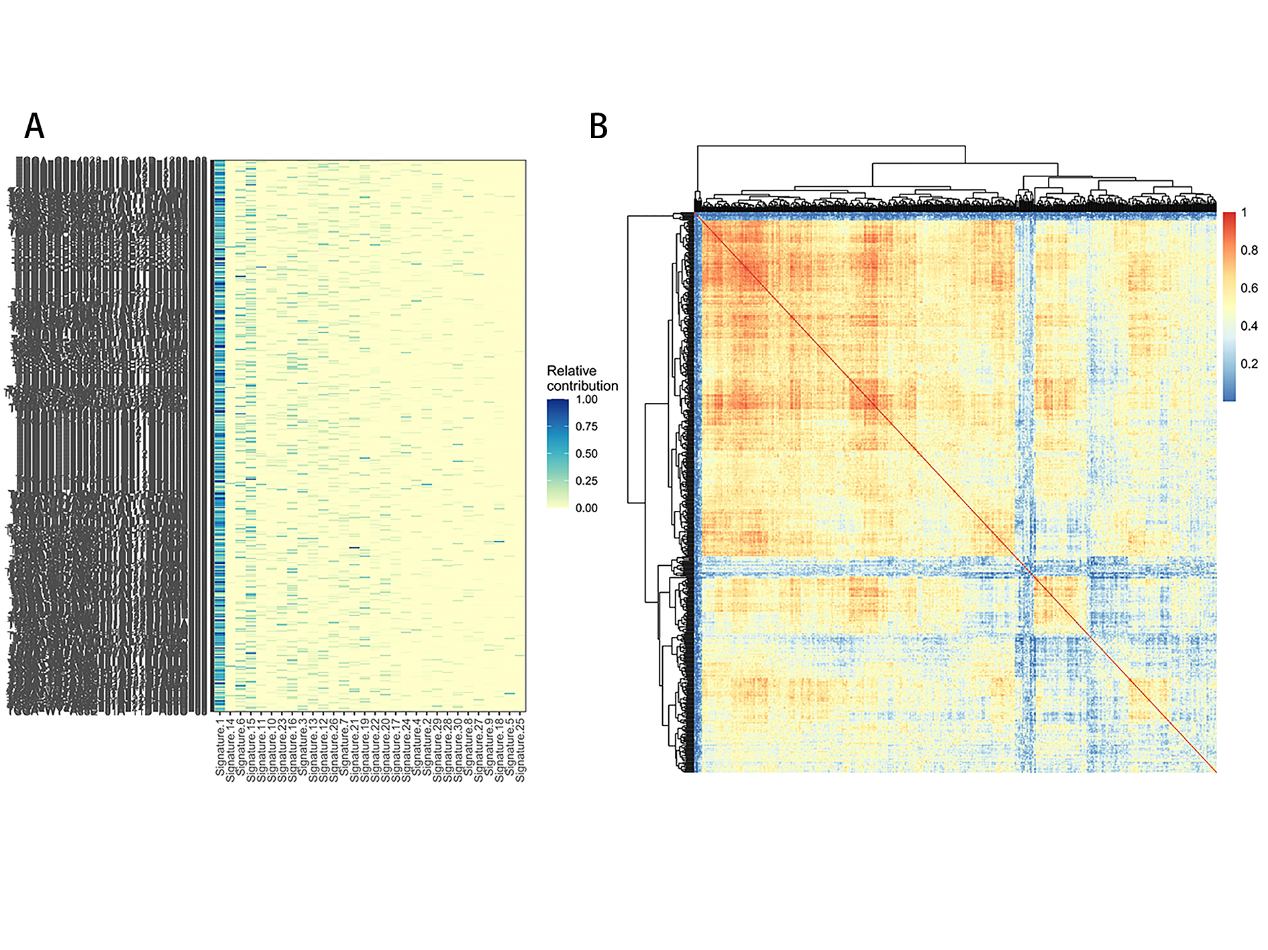
**

**Figure S2 The mutation signatures in each sample.** A COSMIC signatures in LGG cases. B The correlation heat map about the mutation profile in each sample. The red and yellow indicated the higher correlation samples while the blue showed the lower correlation samples.

**
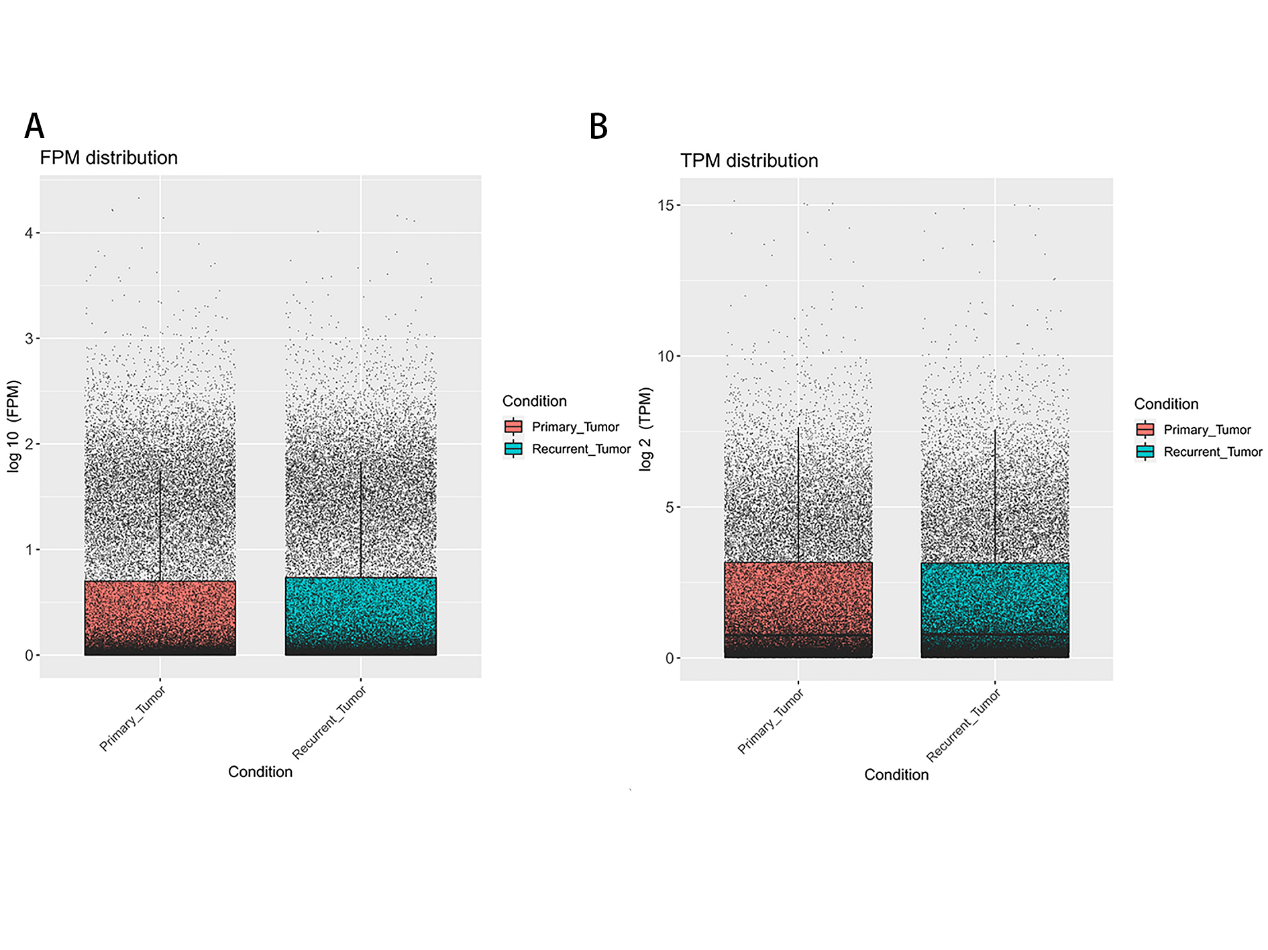
**

**Figure S3. The TPM and FPM of recurrent vs. primary tumors.**

**
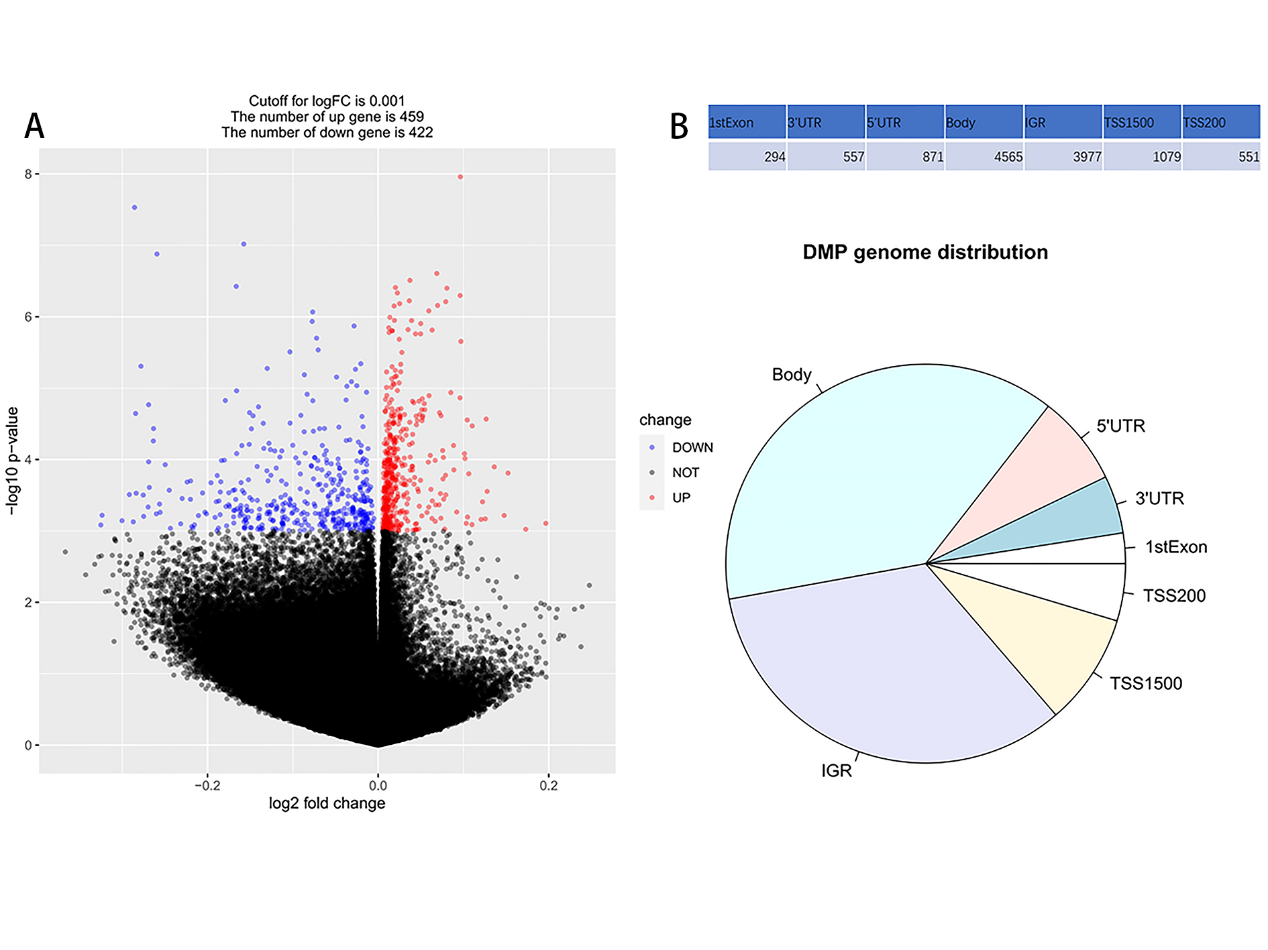
**

**Figure S4. Volcanic plot of differential methylation and the distribution analysis between the primary and recurrent LGG tissues.** A. Volcanic plot of differential methylation. B. The distribution analysis of the differential methylation probes.


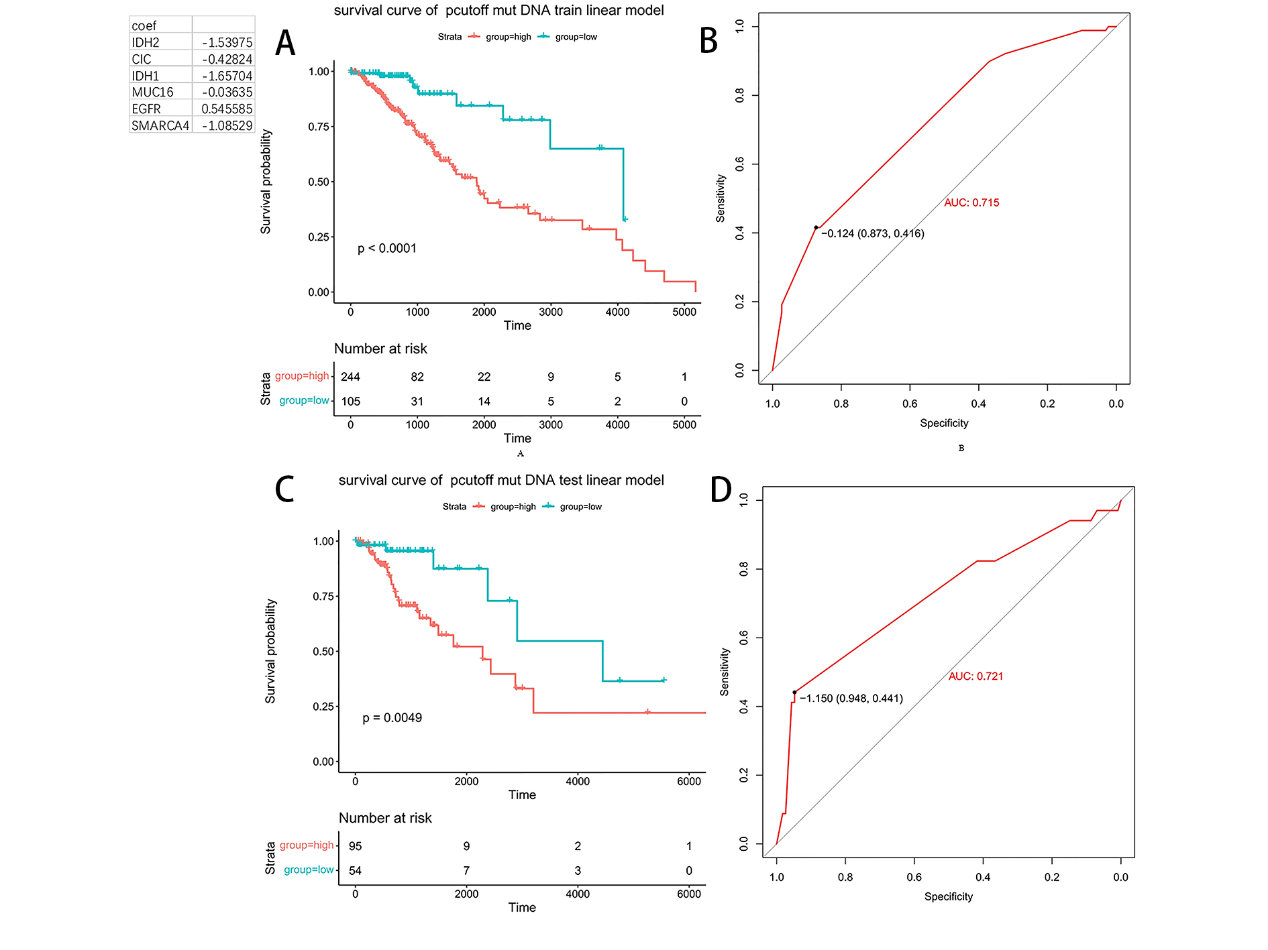


**Figure S5. K-M survival analysis and ROC analysis for 6 frequently mutated genes-based model in the training and testing sets.** A, C. K-M survival analysis in the training (A) and testing (C) sets, respectively. B, D. ROC analysis in the training (B) and testing (D) sets, respectively.


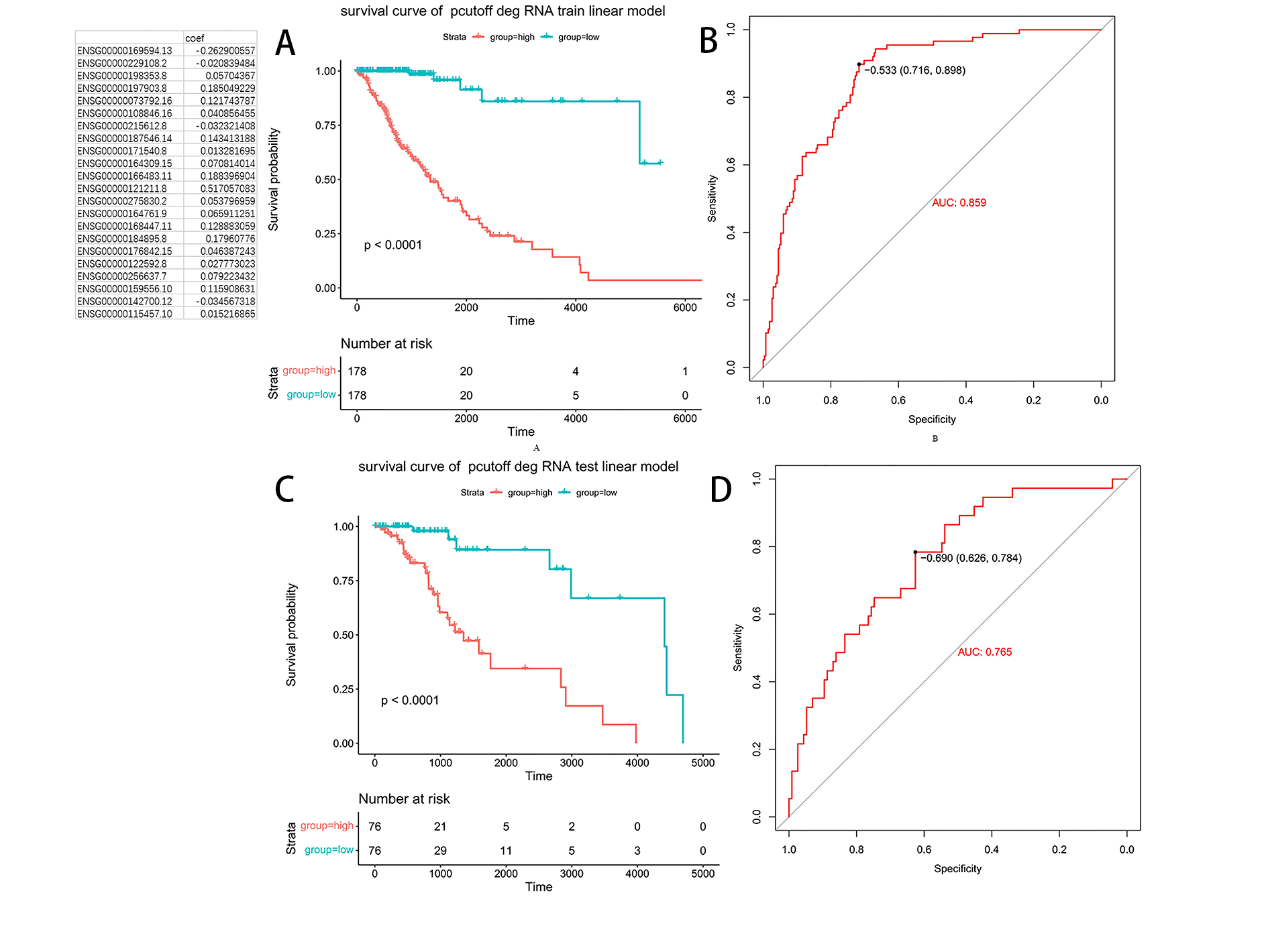


**Figure S6. K-M survival analysis and ROC analysis for 22 DEGs-based model in the training and testing sets.** A, C. K-M survival analysis in the training (A) and testing (C) sets, respectively. B, D. ROC analysis in the training (B) and testing (D) sets, respectively.


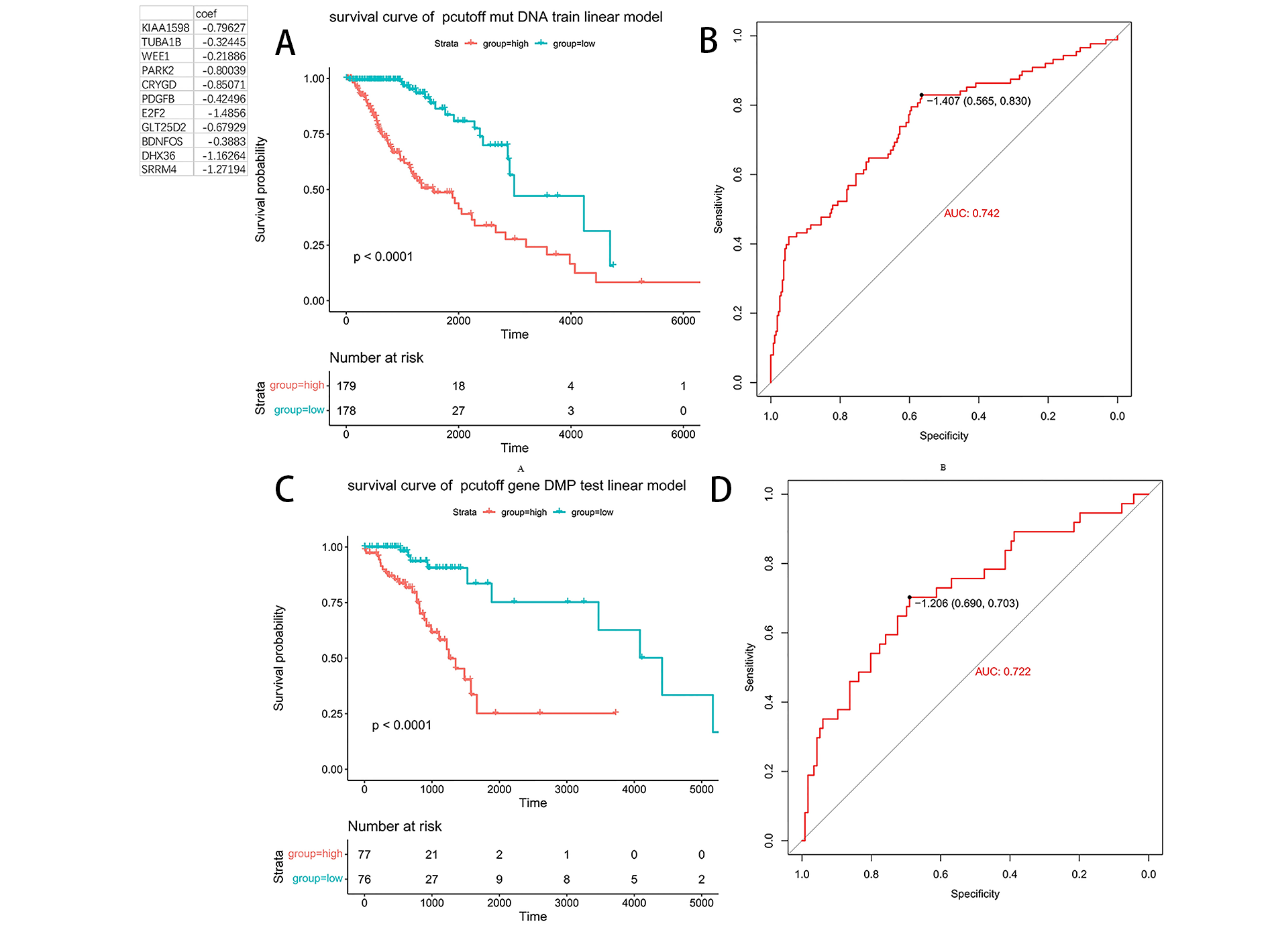


**Figure S7. K-M survival analysis and ROC analysis for 11 DMGs-based model in the training and testing sets.** A, C. K-M survival analysis in the training (A) and testing (C) sets, respectively. B, D. ROC analysis in the training (B) and testing (D) sets, respectively.


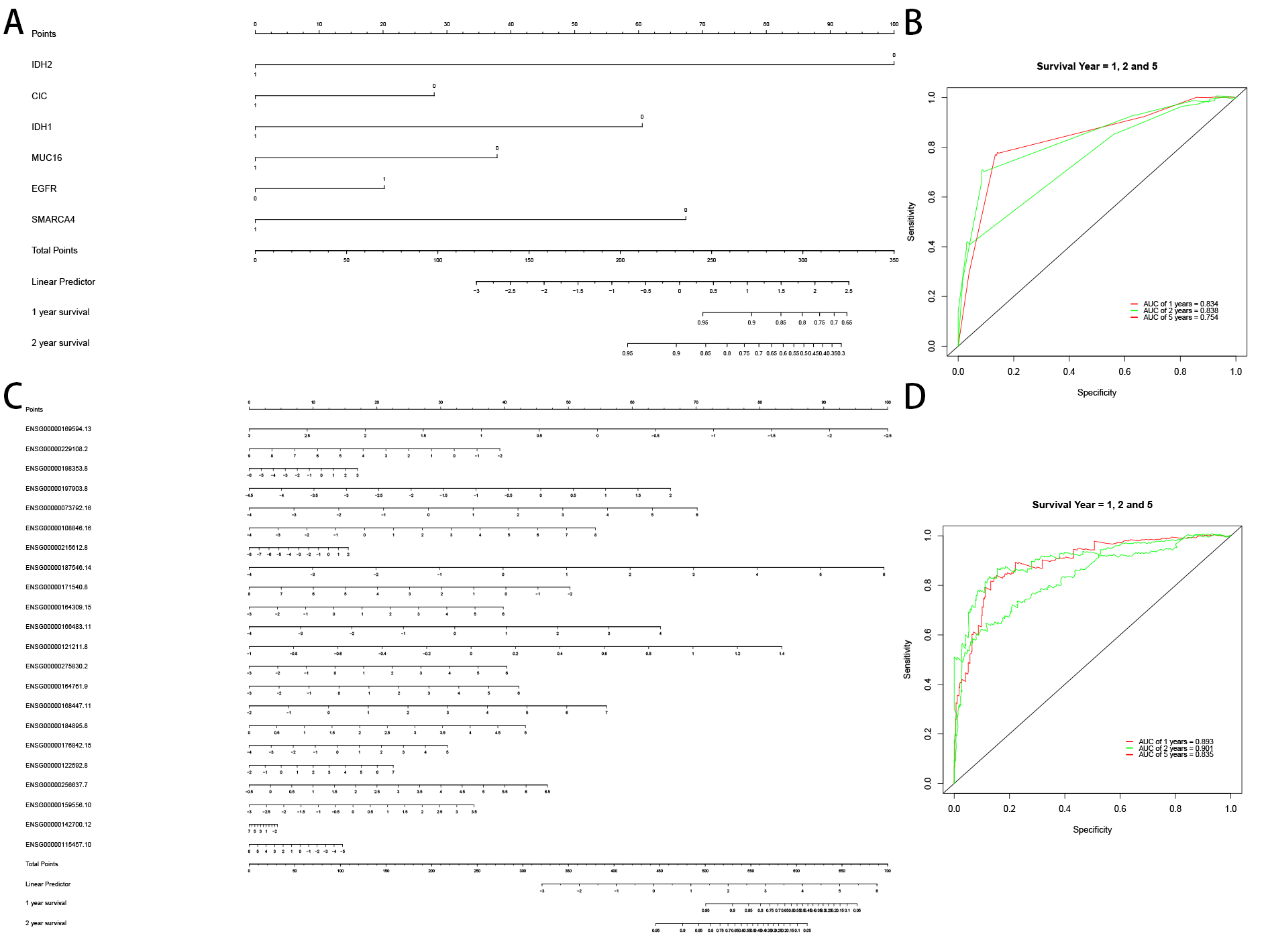

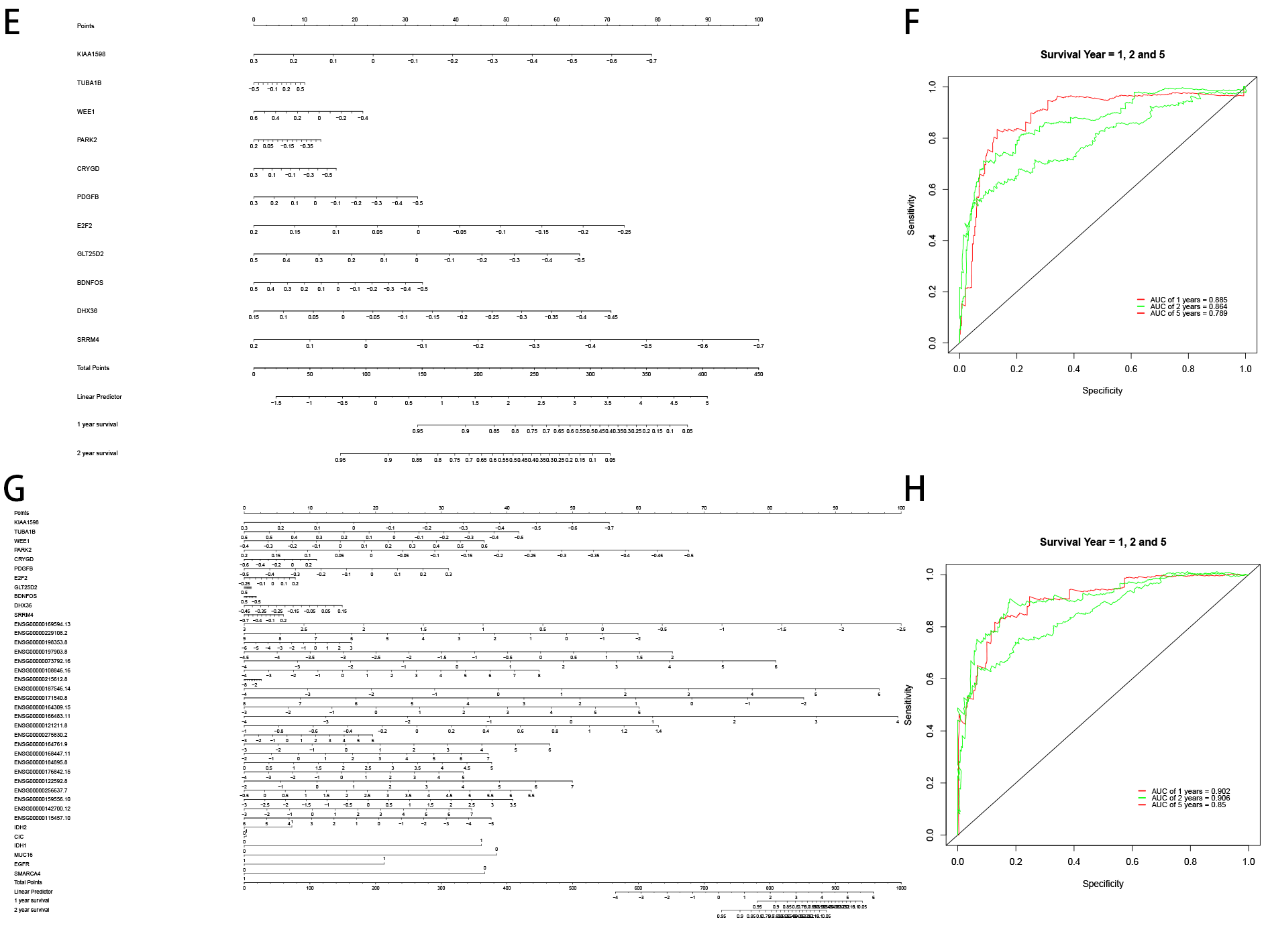


**Figure S8.** A, B nomogram and survial ROC curve of prediction model ustilize WES data. C, D nomogram and survial ROC curve of prediction model ustilize transcriptome data. E, F nomogram and survial ROC curve of prediction model ustilize methylation data. G, H nomogram and survial ROC curve of prediction model ustilize WES, transcriptome and methylation data


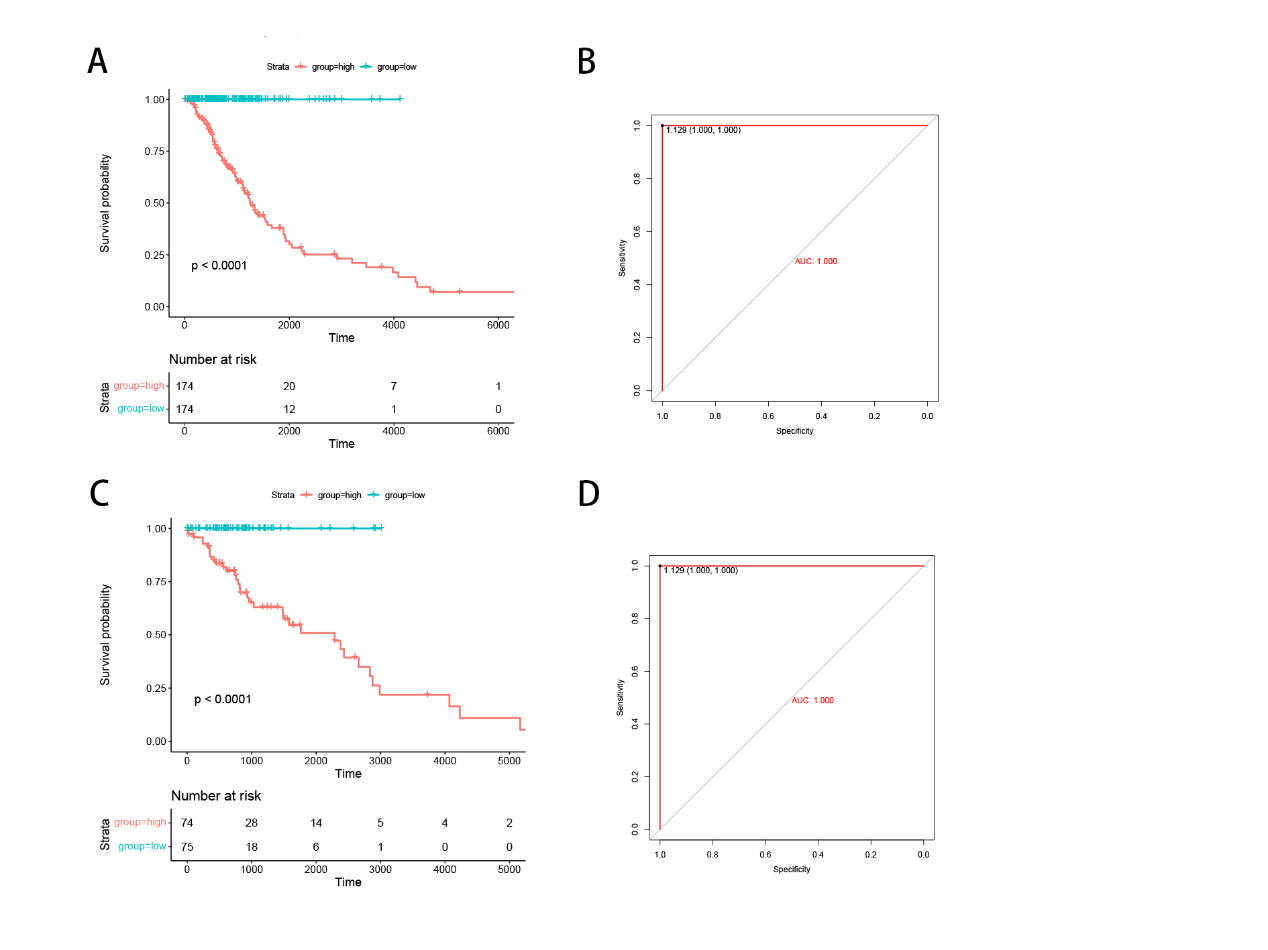


**Figure S9. DeepOmix analysis and ROC analysis for multi-omicsdata in the training and testing sets.** A, C. DeepOmix analysis in the training (A) and testing (C) sets, respectively. B, D. ROC analysis in the training (B) and testing (D) sets, respectively.


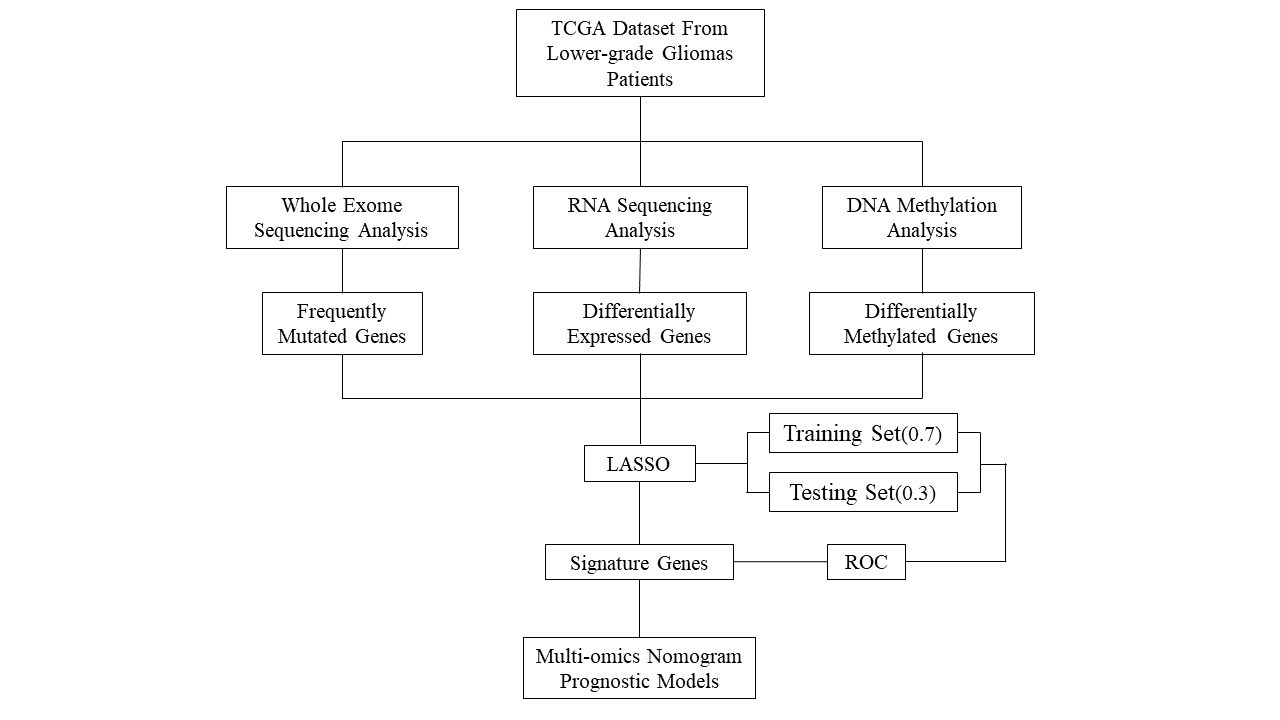


**Figure S10.** Flow chart of the research study.

**Table S1 Significantly up-regulated genes in recurrent vs. primary LGGs (log2FoldChange >2)**

| chr | gene_name | gene_type | padj | log2FoldChange |
| --- | --- | --- | --- | --- |
| chr6 | *C6orf15* | protein_coding | 0.020647 | 4.630677 |
| chr12 | *DAO* | protein_coding | 8.50E-11 | 4.55818 |
| chr7 | *MEOX2* | protein_coding | 2.00E-08 | 4.380905 |
| chr7 | *LINC02587* | lncRNA | 6.22E-06 | 4.266166 |
| chr15 | *AC037471.1* | processed_pseudogene | 3.37E-05 | 4.210297 |
| chr3 | *LTF* | protein_coding | 2.62E-07 | 4.072193 |
| chr18 | *MIR3976HG* | lncRNA | 2.47E-05 | 4.063753 |
| chr12 | *LINC00507* | lncRNA | 1.08E-05 | 3.921326 |
| chr8 | *LINC00588* | lncRNA | 0.001003 | 3.903372 |
| chr1 | *LGR6* | protein_coding | 1.30E-07 | 3.78286 |
| chr12 | *LINC02822* | lncRNA | 0.000116 | 3.667197 |
| chrY | *SRY* | protein_coding | 0.014441 | 3.647781 |
| chr22 | *IGLV8-61* | IG_V_gene | 0.040229 | 3.498763 |
| chr4 | *LINC02475* | lncRNA | 0.000164 | 3.442847 |
| chr6 | *AL355596.2* | lncRNA | 7.60E-05 | 3.411305 |
| chr12 | *TESPA1* | protein_coding | 1.04E-05 | 3.345157 |
| chr18 | *GRAMD4P7* | processed_pseudogene | 0.001809 | 3.320726 |
| chr12 | *KRT6A* | protein_coding | 0.016291 | 3.313301 |
| chr2 | *LINC01876* | lncRNA | 0.003923 | 3.307712 |
| chr20 | *NPBWR2* | protein_coding | 0.004894 | 3.295289 |
| chr12 | *AC126564.1* | TEC | 1.80E-06 | 3.293649 |
| chr22 | *IGLV3-25* | IG_V_gene | 0.031065 | 3.257335 |
| chr17 | *KRT13* | protein_coding | 0.012046 | 3.252798 |
| chr14 | *IGHV3-48* | IG_V_gene | 0.041807 | 3.245706 |
| chr8 | *AC021613.1* | lncRNA | 2.30E-05 | 3.147213 |
| chr10 | *LIPF* | protein_coding | 0.045985 | 3.142065 |
| chr5 | *CTXN3* | protein_coding | 0.000474 | 3.136516 |
| chr12 | *AC025160.1* | lncRNA | 0.014267 | 3.131171 |
| chr1 | *AL450352.1* | lncRNA | 0.010436 | 3.090237 |
| chr5 | *CARTPT* | protein_coding | 0.002573 | 3.043549 |
| chr7 | *LINC01007* | lncRNA | 0.007918 | 3.031537 |
| chr5 | *PCDHGB5* | protein_coding | 3.89E-06 | 3.010299 |
| chr22 | *IGLV2-8* | IG_V_gene | 0.041573 | 3.007072 |
| chr1 | *SMCP* | protein_coding | 0.000137 | 2.997794 |
| chr12 | *SLC17A8* | protein_coding | 3.40E-06 | 2.995641 |
| chr7 | *ITPRID1* | protein_coding | 0.000497 | 2.992869 |
| chr2 | *PAX3* | protein_coding | 0.021307 | 2.982502 |
| chr13 | *LINC01055* | lncRNA | 0.002868 | 2.966434 |
| chr14 | *AL355916.2* | lncRNA | 3.71E-10 | 2.964165 |
| chr22 | *LINC00898* | lncRNA | 0.006861 | 2.953068 |
| chr5 | *AC008708.2* | lncRNA | 0.000578 | 2.920835 |
| chr4 | *AC112236.1* | lncRNA | 0.000101 | 2.917577 |
| chr12 | *LINC02470* | lncRNA | 0.001243 | 2.913919 |
| chr17 | *TEKT1* | protein_coding | 3.37E-07 | 2.9087 |
| chr20 | *KCNS1* | protein_coding | 3.32E-05 | 2.895573 |
| chr5 | *ARPP19P1* | processed_pseudogene | 0.000184 | 2.865375 |
| chr5 | *SHISAL2B* | protein_coding | 0.000194 | 2.860025 |
| chr6 | *PRSS16* | protein_coding | 0.000123 | 2.858637 |
| chr7 | *HOXA7* | protein_coding | 0.021428 | 2.848676 |
| chr13 | *AL158065.1* | lncRNA | 0.000985 | 2.845254 |
| chr7 | *AC092447.5* | lncRNA | 0.003189 | 2.839307 |
| chr11 | *AP003032.1* | lncRNA | 0.036997 | 2.834437 |
| chr18 | *SLC14A2* | protein_coding | 2.54E-07 | 2.832973 |
| chr18 | *DSG3* | protein_coding | 0.006162 | 2.832559 |
| chr18 | *AC023421.1* | lncRNA | 6.20E-07 | 2.81472 |
| chr15 | *ANKRD34C* | protein_coding | 3.97E-05 | 2.773477 |
| chr7 | *AGR3* | protein_coding | 0.039242 | 2.771957 |
| chr2 | *AC079584.1* | lncRNA | 0.001088 | 2.723869 |
| chrX | *DGKK* | protein_coding | 3.40E-06 | 2.716766 |
| chr16 | *AC137056.1* | lncRNA | 0.000704 | 2.715575 |
| chr9 | *AL353732.2* | lncRNA | 0.000353 | 2.706356 |
| chrX | *ESX1* | protein_coding | 0.004106 | 2.705424 |
| chr2 | *MAP3K19* | protein_coding | 6.81E-05 | 2.679884 |
| chr4 | *AC016687.2* | lncRNA | 0.011965 | 2.669102 |
| chr3 | *GMNC* | protein_coding | 0.003136 | 2.667681 |
| chr22 | *IGLV1-40* | IG_V_gene | 0.016491 | 2.648747 |
| chr3 | *LINC02030* | lncRNA | 0.001585 | 2.644065 |
| chr17 | *KRT33A* | protein_coding | 0.000306 | 2.641559 |
| chr12 | *AC068787.1* | TEC | 0.020184 | 2.641407 |
| chr3 | *STAC* | protein_coding | 0.000105 | 2.631004 |
| chr18 | *SLC14A1* | protein_coding | 7.75E-06 | 2.629771 |
| chr2 | *AC092675.2* | lncRNA | 0.009586 | 2.626404 |
| chr2 | *AC018712.1* | processed_pseudogene | 0.001965 | 2.613801 |
| chr10 | *CERNA2* | lncRNA | 0.002407 | 2.612023 |
| chr7 | *COL28A1* | protein_coding | 4.33E-07 | 2.588109 |
| chr6 | *GLP1R* | protein_coding | 2.55E-05 | 2.579206 |
| chr17 | *AC027045.3* | lncRNA | 0.004791 | 2.577954 |
| chr11 | *AC018716.2* | lncRNA | 0.001062 | 2.568743 |
| chrX | *MTND4P24* | processed_pseudogene | 3.20E-05 | 2.564167 |
| chr6 | *HMGB1P13* | processed_pseudogene | 0.000853 | 2.548651 |
| chr8 | *LINC01289* | lncRNA | 0.03235 | 2.533076 |
| chr15 | *TMC3* | protein_coding | 0.00134 | 2.527264 |
| chr7 | *TRGJP2* | TR_J_gene | 0.000686 | 2.526186 |
| chr2 | *CNN2P8* | processed_pseudogene | 0.000953 | 2.523852 |
| chr12 | *LINC02359* | lncRNA | 0.005429 | 2.511257 |
| chr14 | *AL133240.2* | lncRNA | 0.000223 | 2.507161 |
| chr7 | *AC237221.2* | TEC | 0.036467 | 2.506671 |
| chr7 | *GDI2P1* | processed_pseudogene | 0.004766 | 2.487629 |
| chr11 | *SAA1* | protein_coding | 0.026814 | 2.485757 |
| chr14 | *IGHV3-23* | IG_V_gene | 0.028006 | 2.48169 |
| chrX | *AL022151.1* | lncRNA | 0.003499 | 2.476015 |
| chr1 | *LINC02869* | lncRNA | 0.000682 | 2.465795 |
| chr11 | *AP001804.1* | lncRNA | 0.019773 | 2.448103 |
| chr1 | *LCE1E* | protein_coding | 0.000832 | 2.447045 |
| chr17 | *CCL1* | protein_coding | 0.01006 | 2.440922 |
| chr7 | *AC073324.1* | processed_pseudogene | 0.039678 | 2.440641 |
| chr2 | *LINC01965* | lncRNA | 0.026596 | 2.440345 |
| chr22 | *IGLV7-46* | IG_V_gene | 0.045271 | 2.439667 |
| chr7 | *AC005998.1* | lncRNA | 9.34E-05 | 2.43707 |
| chr14 | *IGHV3-15* | IG_V_gene | 0.008738 | 2.436985 |
| chr2 | *RN7SKP83* | misc_RNA | 0.002047 | 2.435746 |
| chr11 | *HTR3B* | protein_coding | 0.001398 | 2.432656 |
| chr10 | *AC069540.2* | lncRNA | 0.037067 | 2.429253 |
| chr6 | *AL590009.1* | lncRNA | 0.017077 | 2.427602 |
| chr1 | *AC104459.1* | lncRNA | 0.000869 | 2.411706 |
| chr4 | *SOWAHB* | protein_coding | 0.000159 | 2.404577 |
| chr3 | *CRB3P1* | processed_pseudogene | 0.033089 | 2.399141 |
| chr4 | *AC097478.4* | lncRNA | 0.005894 | 2.390505 |
| chr1 | *KLHDC7A* | protein_coding | 0.019733 | 2.388604 |
| chr12 | *AC084816.1* | lncRNA | 0.004801 | 2.386884 |
| chr17 | *LINC01982* | lncRNA | 0.00706 | 2.384821 |
| chr10 | *AC244230.2* | lncRNA | 0.014601 | 2.384649 |
| chr18 | *AC090125.2* | lncRNA | 0.028826 | 2.384487 |
| chr14 | *AL158058.1* | lncRNA | 0.000981 | 2.376779 |
| chr4 | *AC104407.1* | lncRNA | 0.000803 | 2.375005 |
| chr7 | *AC011228.2* | lncRNA | 0.006706 | 2.366879 |
| chr18 | *AC006566.2* | lncRNA | 0.000101 | 2.360027 |
| chr13 | *LINC00364* | lncRNA | 0.006868 | 2.357458 |
| chr12 | *LINC02347* | lncRNA | 0.00973 | 2.357274 |
| chr3 | *AC018358.1* | lncRNA | 0.012023 | 2.356895 |
| chr2 | *TFCP2L1* | protein_coding | 6.07E-05 | 2.355053 |
| chr2 | *AL845331.1* | processed_pseudogene | 0.018689 | 2.352767 |
| chr7 | *NEUROD6* | protein_coding | 0.002981 | 2.349788 |
| chr17 | *CD300LG* | protein_coding | 1.01E-05 | 2.348702 |
| chr6 | *AL512658.2* | lncRNA | 0.001021 | 2.336906 |
| chr5 | *ANKRD55* | protein_coding | 1.59E-06 | 2.336724 |
| chr18 | *LINC01894* | lncRNA | 2.27E-06 | 2.327773 |
| chr17 | *ABCC3* | protein_coding | 0.00092 | 2.326523 |
| chr3 | *LINC01994* | lncRNA | 0.003242 | 2.324788 |
| chr6 | *MAS1* | protein_coding | 0.002721 | 2.318578 |
| chr5 | *AC008459.1* | lncRNA | 0.008913 | 2.316502 |
| chr8 | *LINC01602* | lncRNA | 0.020488 | 2.314941 |
| chr4 | *AC005699.1* | lncRNA | 0.001481 | 2.308394 |
| chr2 | *AC110754.1* | processed_pseudogene | 0.009984 | 2.30159 |
| chrX | *IGSF1* | protein_coding | 5.09E-07 | 2.299442 |
| chr22 | *IGLV1-47* | IG_V_gene | 0.003696 | 2.298958 |
| chr7 | *AC007652.3* | lncRNA | 0.008658 | 2.298814 |
| chr8 | *LINC01414* | lncRNA | 0.006875 | 2.297385 |
| chr10 | *ARMC3* | protein_coding | 0.00013 | 2.292391 |
| chr17 | *AC104024.2* | lncRNA | 0.003099 | 2.284661 |
| chr13 | *LINC02343* | lncRNA | 0.000143 | 2.276919 |
| chr9 | *AL157886.1* | lncRNA | 0.000725 | 2.267628 |
| chr12 | *LMNTD1* | protein_coding | 1.58E-06 | 2.264692 |
| chr2 | *IGKV3-20* | IG_V_gene | 0.036036 | 2.263827 |
| chr17 | *AC004147.3* | lncRNA | 0.010586 | 2.260861 |
| chr10 | *SFTPA1* | protein_coding | 0.012831 | 2.260673 |
| chr2 | *ZNF385B* | protein_coding | 1.34E-06 | 2.258285 |
| chr21 | *AL773573.1* | lncRNA | 0.032533 | 2.256666 |
| chr8 | *AC100872.2* | lncRNA | 0.046226 | 2.256577 |
| chr18 | *AC007922.4* | lncRNA | 0.047945 | 2.255892 |
| chr10 | *AL450322.2* | lncRNA | 0.00917 | 2.255189 |
| chr5 | *AC026780.1* | lncRNA | 2.30E-05 | 2.249929 |
| chr22 | *IGLV2-14* | IG_V_gene | 0.04121 | 2.247412 |
| chrX | *AL513487.1* | lncRNA | 0.011095 | 2.247277 |
| chr15 | *ANKRD34C-AS1* | lncRNA | 0.001256 | 2.245895 |
| chr13 | *CLYBL-AS1* | lncRNA | 0.009136 | 2.244819 |
| chr20 | *LINC01721* | lncRNA | 0.024431 | 2.231183 |
| chr22 | *AL020994.2* | lncRNA | 0.011107 | 2.228723 |
| chr15 | *AC104574.1* | lncRNA | 0.017694 | 2.226447 |
| chr4 | *AC097450.1* | lncRNA | 0.001081 | 2.226058 |
| chr8 | *AP006248.3* | TEC | 0.004531 | 2.223565 |
| chr14 | *LINC02301* | lncRNA | 0.005099 | 2.223552 |
| chr14 | *AL133240.1* | lncRNA | 0.021747 | 2.220918 |
| chr14 | *AL133166.1* | lncRNA | 0.001287 | 2.214466 |
| chr10 | *AC245041.2* | lncRNA | 0.015012 | 2.209543 |
| chr2 | *LINC01854* | lncRNA | 0.008015 | 2.208872 |
| chr4 | *AC079298.3* | lncRNA | 1.05E-06 | 2.203066 |
| chr4 | *LINC00499* | lncRNA | 0.000399 | 2.199988 |
| chr6 | *AL355596.1* | lncRNA | 0.002173 | 2.19577 |
| chr18 | *AC023421.2* | lncRNA | 0.000578 | 2.186176 |
| chr7 | *AC004022.1* | processed_pseudogene | 0.003853 | 2.178084 |
| chr20 | *AL049541.1* | lncRNA | 0.006406 | 2.176829 |
| chr5 | *CCT7P2* | processed_pseudogene | 3.24E-05 | 2.175343 |
| chr3 | *RPS8P6* | processed_pseudogene | 0.026138 | 2.16871 |
| chr7 | *AC084864.2* | lncRNA | 0.028672 | 2.163669 |
| chr13 | *AL512452.1* | lncRNA | 0.002352 | 2.163555 |
| chr20 | *HAO1* | protein_coding | 0.038107 | 2.156327 |
| chrX | *EFHC2* | protein_coding | 1.06E-05 | 2.155266 |
| chr19 | *AC010641.2* | lncRNA | 0.001601 | 2.146748 |
| chr7 | *AC005999.2* | lncRNA | 1.58E-06 | 2.145637 |
| chr9 | *SOHLH1* | protein_coding | 0.00092 | 2.141837 |
| chr2 | *DRC1* | protein_coding | 0.00334 | 2.140261 |
| chr22 | *AP000357.2* | lncRNA | 0.015805 | 2.136865 |
| chr4 | *GBA3* | polymorphic_pseudogene | 0.003908 | 2.134607 |
| chr5 | *HTR1A* | protein_coding | 0.00046 | 2.128352 |
| chr13 | *FAM216B* | protein_coding | 0.040339 | 2.12424 |
| chr4 | *FGFBP2* | protein_coding | 8.87E-05 | 2.123481 |
| chr8 | *LINC01606* | lncRNA | 0.005708 | 2.117705 |
| chr5 | *SLC6A7* | protein_coding | 0.003891 | 2.117022 |
| chr18 | *AC011139.1* | lncRNA | 0.000996 | 2.106226 |
| chr8 | *AF228727.1* | lncRNA | 0.012812 | 2.1055 |
| chr11 | *LINC02690* | lncRNA | 0.000292 | 2.100657 |
| chr5 | *LINC01485* | lncRNA | 1.37E-05 | 2.09891 |
| chrX | *AF241726.1* | processed_pseudogene | 0.040887 | 2.097695 |
| chr10 | *LINC00842* | lncRNA | 0.001504 | 2.095943 |
| chrX | *PIH1D3* | protein_coding | 0.047037 | 2.094927 |
| chr16 | *ZSCAN10* | protein_coding | 0.000843 | 2.091668 |
| chr13 | *RNA5SP39* | rRNA_pseudogene | 0.008893 | 2.083659 |
| chr10 | *AL356157.3* | lncRNA | 0.043284 | 2.081746 |
| chr14 | *NGB* | protein_coding | 0.00441 | 2.080119 |
| chr17 | *AC002546.1* | lncRNA | 0.000206 | 2.07312 |
| chr7 | *HTR5A* | protein_coding | 0.00177 | 2.070437 |
| chr5 | *AC122697.1* | lncRNA | 0.022319 | 2.064267 |
| chr6 | *AL033519.5* | lncRNA | 0.000143 | 2.063467 |
| chr9 | *AL353753.1* | lncRNA | 0.002956 | 2.062838 |
| chr1 | *LMX1A* | protein_coding | 0.034075 | 2.058088 |
| chr1 | *AL353709.1* | lncRNA | 0.006006 | 2.054516 |
| chr15 | *AC026992.1* | lncRNA | 0.002555 | 2.054221 |
| chr14 | *IGHA2* | IG_C_gene | 0.023346 | 2.053867 |
| chr1 | *TNNT2* | protein_coding | 0.020188 | 2.053814 |
| chr12 | *AC084880.4* | lncRNA | 0.025487 | 2.051077 |
| chr4 | *DDIT4L* | protein_coding | 0.00269 | 2.04971 |
| chr4 | *AC097709.1* | lncRNA | 7.24E-05 | 2.04729 |
| chr9 | *AL583827.1* | lncRNA | 0.012688 | 2.046808 |
| chr20 | *LINC01726* | lncRNA | 0.001193 | 2.044904 |
| chr7 | *AC021218.1* | lncRNA | 0.020519 | 2.040959 |
| chrX | *SMPX* | protein_coding | 0.006926 | 2.04045 |
| chr2 | *AC016682.1* | lncRNA | 0.00011 | 2.037908 |
| chr2 | *AC007463.2* | lncRNA | 9.67E-05 | 2.037189 |
| chr10 | *AL133482.1* | lncRNA | 1.10E-05 | 2.035526 |
| chr7 | *AC004899.1* | processed_pseudogene | 0.000458 | 2.033402 |
| chr11 | *AC103855.2* | lncRNA | 0.002021 | 2.025009 |
| chr12 | *AC078922.1* | lncRNA | 0.010379 | 2.021586 |
| chr7 | *AC005999.1* | lncRNA | 7.58E-05 | 2.020786 |
| chr1 | *IVL* | protein_coding | 0.013821 | 2.0203 |
| chr6 | *LINC02518* | lncRNA | 0.00525 | 2.016329 |
| chr1 | *AL592182.2* | lncRNA | 0.011828 | 2.016172 |
| chr14 | IGHA1 | IG_C_gene | 0.007665 | 2.014949 |
| chr1 | AL603840.1 | lncRNA | 5.89E-05 | 2.008431 |
| chr3 | PRICKLE2-AS2 | lncRNA | 0.005744 | 2.002566 |

**Table S2 Significantly down-regulated genes in recurrent vs. primary LGGs (log2FoldChange >2)**

| chr | gene_name | gene_type | pvalue | padj | log2FoldChange |
| --- | --- | --- | --- | --- | --- |
| chr1 | DMBX1 | protein_coding | 5.99E-24 | 4.28E-20 | -6.35802 |
| chrX | GPR50 | protein_coding | 4.95E-20 | 1.18E-16 | -5.47617 |
| chr10 | HMX2 | protein_coding | 4.51E-07 | 2.82E-05 | -5.45513 |
| chr8 | MAFA | protein_coding | 7.84E-30 | 1.40E-25 | -5.44083 |
| chr12 | COL2A1 | protein_coding | 1.86E-26 | 2.21E-22 | -5.21325 |
| chr5 | TLX3 | protein_coding | 2.20E-06 | 9.78E-05 | -4.9946 |
| chrX | KC877982.1 | lncRNA | 5.58E-11 | 1.63E-08 | -4.81181 |
| chr9 | BARX1 | protein_coding | 5.74E-16 | 8.19E-13 | -4.66951 |
| chr10 | LBX1-AS1 | lncRNA | 1.09E-07 | 9.18E-06 | -4.58941 |
| chr17 | MEOX1 | protein_coding | 1.26E-22 | 5.62E-19 | -4.58713 |
| chr6 | AL596442.3 | lncRNA | 6.32E-07 | 3.68E-05 | -4.41299 |
| chr1 | HES2 | protein_coding | 1.75E-20 | 5.19E-17 | -4.39407 |
| chr11 | IGF2 | protein_coding | 5.15E-18 | 9.66E-15 | -4.34332 |
| chr19 | ONECUT3 | protein_coding | 1.14E-23 | 6.78E-20 | -4.31578 |
| chr15 | BNC1 | protein_coding | 1.81E-24 | 1.62E-20 | -4.30001 |
| chr16 | AC120498.10 | lncRNA | 1.05E-17 | 1.74E-14 | -4.26164 |
| chr11 | LMNTD2-AS1 | lncRNA | 1.30E-30 | 4.62E-26 | -4.22031 |
| chr7 | HOXA11-AS | lncRNA | 1.90E-06 | 8.69E-05 | -4.15596 |
| chr5 | PITX1 | protein_coding | 8.39E-14 | 5.65E-11 | -4.06828 |
| chr11 | IGF2-AS | lncRNA | 4.33E-12 | 1.86E-09 | -3.94495 |
| chr8 | AC078906.1 | lncRNA | 3.37E-14 | 2.56E-11 | -3.82688 |
| chr17 | AC209154.1 | lncRNA | 0.001841 | 0.015912 | -3.79437 |
| chr6 | H2AC13 | protein_coding | 1.33E-15 | 1.52E-12 | -3.75098 |
| chr17 | ALOX15 | protein_coding | 2.76E-16 | 4.10E-13 | -3.66054 |
| chr9 | AL136981.1 | transcribed_unprocessed_pseudogene | 3.88E-10 | 9.28E-08 | -3.57385 |
| chr2 | COL3A1 | protein_coding | 9.45E-14 | 6.24E-11 | -3.54671 |
| chr20 | PRND | protein_coding | 2.52E-12 | 1.18E-09 | -3.4934 |
| chr17 | COL1A1 | protein_coding | 2.04E-12 | 9.83E-10 | -3.40246 |
| chr12 | AC126175.2 | lncRNA | 1.41E-12 | 7.07E-10 | -3.37981 |
| chrX | AC004835.1 | lncRNA | 1.49E-14 | 1.33E-11 | -3.35921 |
| chr12 | NTF3 | protein_coding | 3.12E-12 | 1.39E-09 | -3.35239 |
| chr17 | HOXB8 | protein_coding | 0.001643 | 0.014601 | -3.34094 |
| chr7 | AC004080.2 | lncRNA | 2.56E-05 | 0.000646 | -3.3286 |
| chr18 | GATA6-AS1 | lncRNA | 3.42E-07 | 2.29E-05 | -3.32685 |
| chr4 | NKX3-2 | protein_coding | 3.90E-10 | 9.28E-08 | -3.21743 |
| chr9 | ROR2 | protein_coding | 2.92E-14 | 2.32E-11 | -3.20666 |
| chr22 | TBX1 | protein_coding | 1.22E-19 | 2.72E-16 | -3.19596 |
| chr8 | EBF2 | protein_coding | 2.49E-15 | 2.61E-12 | -3.19553 |
| chr6 | H3C10 | protein_coding | 3.53E-20 | 9.00E-17 | -3.18056 |
| chr19 | COMP | protein_coding | 6.35E-11 | 1.81E-08 | -3.17935 |
| chr8 | MAFA-AS1 | lncRNA | 4.60E-05 | 0.000999 | -3.15299 |
| chr12 | AC026310.2 | lncRNA | 8.85E-11 | 2.48E-08 | -3.11331 |
| chr1 | MFAP2 | protein_coding | 1.56E-11 | 5.46E-09 | -3.08207 |
| chr9 | COL5A1 | protein_coding | 4.12E-12 | 1.79E-09 | -3.04899 |
| chr12 | HOXC5 | protein_coding | 0.00033 | 0.00441 | -3.00602 |
| chr13 | CDX2 | protein_coding | 4.94E-07 | 3.03E-05 | -2.9916 |
| chr16 | GP2 | protein_coding | 0.001194 | 0.011494 | -2.96618 |
| chr20 | MMP9 | protein_coding | 8.20E-07 | 4.51E-05 | -2.96502 |
| chr7 | MNX1 | protein_coding | 1.34E-08 | 1.62E-06 | -2.94743 |
| chr16 | SLC22A31 | protein_coding | 5.12E-09 | 7.58E-07 | -2.92309 |
| chr17 | HES7 | protein_coding | 2.25E-20 | 6.18E-17 | -2.91288 |
| chr12 | HOXC8 | protein_coding | 0.00401 | 0.028645 | -2.87533 |
| chr5 | IRX4-AS1 | lncRNA | 0.002394 | 0.019468 | -2.85737 |
| chr19 | KLK2 | protein_coding | 0.005755 | 0.036967 | -2.85109 |
| chr15 | CRABP1 | protein_coding | 8.49E-07 | 4.65E-05 | -2.84891 |
| chr11 | LMNTD2 | protein_coding | 4.85E-23 | 2.47E-19 | -2.80738 |
| chr14 | LINC00520 | lncRNA | 5.38E-09 | 7.90E-07 | -2.77389 |
| chr2 | WNT6 | protein_coding | 1.08E-17 | 1.74E-14 | -2.77137 |
| chr12 | HOXC9 | protein_coding | 0.007643 | 0.045237 | -2.75277 |
| chr8 | AC105118.1 | lncRNA | 0.000367 | 0.004752 | -2.69842 |
| chr17 | TBX21 | protein_coding | 1.18E-15 | 1.40E-12 | -2.68231 |
| chr22 | ZNF280A | protein_coding | 8.54E-05 | 0.001601 | -2.67827 |
| chr17 | IGF2BP1 | protein_coding | 2.24E-06 | 9.92E-05 | -2.67823 |
| chr20 | AL031674.1 | lncRNA | 0.000205 | 0.003089 | -2.67753 |
| chr1 | FOXD2 | protein_coding | 5.22E-14 | 3.80E-11 | -2.6757 |
| chr2 | LINC01614 | lncRNA | 1.80E-05 | 0.000498 | -2.67444 |
| chr15 | ADAMTS7 | protein_coding | 1.11E-15 | 1.37E-12 | -2.6666 |
| chr1 | NHLH1 | protein_coding | 1.95E-14 | 1.66E-11 | -2.65214 |
| chr2 | SIX2 | protein_coding | 6.02E-11 | 1.73E-08 | -2.64146 |
| chr16 | TPSG1 | protein_coding | 6.09E-14 | 4.26E-11 | -2.63121 |
| chr11 | WNT11 | protein_coding | 4.04E-21 | 1.31E-17 | -2.62879 |
| chr17 | HOXB9 | protein_coding | 0.000744 | 0.008015 | -2.62071 |
| chr7 | AC092685.1 | processed_pseudogene | 0.000271 | 0.003817 | -2.58993 |
| chr2 | COL6A3 | protein_coding | 3.29E-09 | 5.26E-07 | -2.58585 |
| chr17 | AC243773.1 | lncRNA | 0.001335 | 0.012476 | -2.57239 |
| chr2 | HOXD9 | protein_coding | 0.000446 | 0.005456 | -2.57141 |
| chr11 | CD248 | protein_coding | 2.80E-12 | 1.30E-09 | -2.56623 |
| chr13 | SHISA2 | protein_coding | 1.88E-13 | 1.12E-10 | -2.56193 |
| chr21 | COL6A2 | protein_coding | 1.70E-09 | 3.15E-07 | -2.54962 |
| chrX | EGFL6 | protein_coding | 4.23E-09 | 6.49E-07 | -2.53971 |
| chr17 | LHX1-DT | lncRNA | 3.38E-05 | 0.000795 | -2.53458 |
| chr16 | PRR35 | protein_coding | 6.93E-05 | 0.001361 | -2.51089 |
| chr8 | AC090136.3 | lncRNA | 3.23E-07 | 2.20E-05 | -2.50155 |
| chr5 | LINC02104 | lncRNA | 3.39E-10 | 8.35E-08 | -2.49756 |
| chr6 | H2AC11 | protein_coding | 4.83E-12 | 2.03E-09 | -2.48303 |
| chr5 | TRIM7 | protein_coding | 3.04E-21 | 1.09E-17 | -2.47892 |
| chr1 | TNNI1 | protein_coding | 4.41E-07 | 2.79E-05 | -2.4621 |
| chr2 | FAR2P1 | transcribed_unprocessed_pseudogene | 9.65E-07 | 5.17E-05 | -2.45223 |
| chr20 | LINC01524 | lncRNA | 1.93E-06 | 8.81E-05 | -2.44773 |
| chr14 | PAX9 | protein_coding | 2.71E-11 | 8.55E-09 | -2.4475 |
| chr10 | KAZALD1 | protein_coding | 9.30E-16 | 1.23E-12 | -2.43226 |
| chr17 | LHX1 | protein_coding | 6.19E-05 | 0.001251 | -2.41909 |
| chr10 | VAX1 | protein_coding | 2.77E-08 | 2.96E-06 | -2.41569 |
| chr19 | SPC24 | protein_coding | 1.28E-10 | 3.41E-08 | -2.40361 |
| chr9 | PAEP | protein_coding | 0.006103 | 0.038558 | -2.39671 |
| chr14 | NID2 | protein_coding | 5.14E-13 | 2.78E-10 | -2.38067 |
| chr7 | HOXA11 | protein_coding | 0.007691 | 0.045422 | -2.37163 |
| chr17 | NGFR | protein_coding | 1.49E-08 | 1.76E-06 | -2.36027 |
| chr22 | MMP11 | protein_coding | 2.82E-14 | 2.29E-11 | -2.30831 |
| chr2 | AC007240.1 | snoRNA | 0.000346 | 0.004556 | -2.30629 |
| chr9 | COL15A1 | protein_coding | 4.03E-09 | 6.30E-07 | -2.2939 |
| chr12 | HOXC4 | protein_coding | 9.06E-08 | 7.84E-06 | -2.2935 |
| chr16 | SLC6A10P | transcribed_unprocessed_pseudogene | 1.10E-09 | 2.22E-07 | -2.28046 |
| chr14 | NKX2-1-AS1 | lncRNA | 0.00358 | 0.026316 | -2.27949 |
| chr3 | IGF2BP2 | protein_coding | 1.33E-05 | 0.000395 | -2.26646 |
| chr19 | CILP2 | protein_coding | 1.09E-16 | 1.68E-13 | -2.26575 |
| chr22 | SHISA8 | protein_coding | 1.98E-09 | 3.52E-07 | -2.26517 |
| chr7 | LINC01447 | lncRNA | 0.00054 | 0.006285 | -2.26396 |
| chr5 | ADAMTS2 | protein_coding | 1.35E-13 | 8.50E-11 | -2.26187 |
| chr19 | RNASEH2A | protein_coding | 1.23E-14 | 1.12E-11 | -2.24486 |
| chr8 | SCX | protein_coding | 1.71E-11 | 5.81E-09 | -2.23419 |
| chr15 | RNU5A-1 | snRNA | 1.79E-08 | 2.06E-06 | -2.23204 |
| chr20 | MYBL2 | protein_coding | 1.05E-06 | 5.51E-05 | -2.22562 |
| chr2 | SP9 | protein_coding | 2.74E-06 | 0.000116 | -2.2187 |
| chr7 | MNX1-AS1 | lncRNA | 0.00033 | 0.004411 | -2.21761 |
| chr6 | IRF4 | protein_coding | 1.70E-11 | 5.81E-09 | -2.19288 |
| chrX | GPR50-AS1 | lncRNA | 0.002664 | 0.021005 | -2.17787 |
| chr12 | NXPH4 | protein_coding | 1.91E-10 | 4.82E-08 | -2.17105 |
| chr7 | HOTTIP | lncRNA | 0.004627 | 0.031593 | -2.16148 |
| chr2 | HOXD-AS2 | lncRNA | 0.000217 | 0.003218 | -2.15866 |
| chr15 | ADAMTS7P3 | transcribed_unprocessed_pseudogene | 9.81E-11 | 2.71E-08 | -2.151 |
| chr8 | AC103718.1 | lncRNA | 4.83E-05 | 0.001033 | -2.1497 |
| chr17 | AURKB | protein_coding | 3.99E-07 | 2.55E-05 | -2.14338 |
| chr6 | AL031058.1 | lncRNA | 1.75E-10 | 4.48E-08 | -2.13879 |
| chr16 | HSD11B2 | protein_coding | 8.94E-19 | 1.77E-15 | -2.13209 |
| chr2 | AC007402.2 | lncRNA | 2.22E-06 | 9.85E-05 | -2.12695 |
| chr12 | KRT79 | protein_coding | 0.000468 | 0.00565 | -2.12203 |
| chr21 | AL133492.1 | lncRNA | 2.98E-09 | 4.83E-07 | -2.11944 |
| chr12 | LRCOL1 | protein_coding | 5.44E-12 | 2.26E-09 | -2.10366 |
| chr14 | DHRS2 | protein_coding | 4.27E-06 | 0.000164 | -2.10183 |
| chr20 | TFAP2C | protein_coding | 2.38E-07 | 1.67E-05 | -2.09643 |
| chr14 | FOXA1 | protein_coding | 0.001572 | 0.014125 | -2.08916 |
| chr17 | AC025627.1 | lncRNA | 1.65E-06 | 7.81E-05 | -2.08297 |
| chr19 | ACP5 | protein_coding | 8.61E-08 | 7.58E-06 | -2.07378 |
| chr1 | H2BU2P | unitary_pseudogene | 0.000216 | 0.003209 | -2.07281 |
| chr7 | COL1A2 | protein_coding | 4.24E-09 | 6.49E-07 | -2.06761 |
| chr10 | CHAT | protein_coding | 0.005763 | 0.036997 | -2.06756 |
| chr9 | AL354861.3 | lncRNA | 7.32E-12 | 2.97E-09 | -2.0673 |
| chr13 | ANKRD26P3 | unprocessed_pseudogene | 1.45E-05 | 0.000422 | -2.06407 |
| chr8 | SFRP1 | protein_coding | 3.35E-09 | 5.33E-07 | -2.05712 |
| chr12 | AC131212.4 | TEC | 1.22E-10 | 3.31E-08 | -2.05254 |
| chr11 | PHLDA2 | protein_coding | 8.02E-06 | 0.000264 | -2.0397 |
| chr5 | ESM1 | protein_coding | 0.000733 | 0.00792 | -2.03766 |
| chr6 | AL021978.1 | lncRNA | 2.31E-07 | 1.64E-05 | -2.03476 |
| chr6 | GRM4 | protein_coding | 5.05E-07 | 3.09E-05 | -2.03081 |
| chr17 | OTOP2 | protein_coding | 4.50E-05 | 0.000987 | -2.03068 |
| chr6 | FRMD1 | protein_coding | 3.50E-07 | 2.32E-05 | -2.02844 |
| chr17 | DLX3 | protein_coding | 2.86E-06 | 0.00012 | -2.02752 |
| chr15 | AC087481.2 | unprocessed_pseudogene | 0.00036 | 0.004688 | -2.02268 |
| chr12 | HCAR3 | protein_coding | 0.000607 | 0.006877 | -2.02016 |
| chr9 | NR5A1 | protein_coding | 3.73E-07 | 2.44E-05 | -2.01899 |
| chr12 | RNU6-558P | snRNA | 6.72E-05 | 0.001329 | -2.01761 |
| chr11 | AP003068.4 | TEC | 9.58E-18 | 1.71E-14 | -2.01753 |
| chr19 | COX6B2 | protein_coding | 1.01E-11 | 3.99E-09 | -2.01122 |
| chr12 | AC141557.1 | unprocessed_pseudogene | 2.28E-05 | 0.000592 | -2.0105 |
| chr10 | HMX3 | protein_coding | 0.001441 | 0.013225 | -2.00812 |
| chr12 | AC024884.2 | lncRNA | 1.04E-05 | 0.000324 | -2.00793 |
| chr7 | ASB10 | protein_coding | 0.000908 | 0.009348 | -2.00386 |
